# Epigenetics of Autism Spectrum Disorders: A Multi-level Analysis Combining Epi-signature, Age Acceleration, Epigenetic Drift and Rare Epivariations Using Public Datasets

**DOI:** 10.2174/1570159X21666230725142338

**Published:** 2023-09-01

**Authors:** Gentilini Davide, Cavagnola Rebecca, Possenti Irene, Calzari Luciano, Ranucci Francesco, Nola Marta, Olivola Miriam, Brondino Natascia, Politi Pierluigi

**Affiliations:** 1 Department of Brain and Behavioral Sciences, University of Pavia, Pavia, 27100, Italy;; 2 Bioinformatics and Statistical Genomics Unit, IRCCS Istituto Auxologico Italiano, Milan, 20090, Italy;; 3 Department of Statistical Sciences Paolo Fortunati, University of Bologna, Bologna, Italy

**Keywords:** Autism spectrum disorders, epigenetics, epigenetic drift, epivariations, age acceleration, blood, brain

## Abstract

**Background:**

Epigenetics of Autism Spectrum Disorders (ASD) is still an understudied field. The majority of the studies on the topic used an approach based on mere classification of cases and controls.

**Objective:**

The present study aimed at providing a multi-level approach in which different types of epigenetic analysis (epigenetic drift, age acceleration) are combined.

**Methods:**

We used publicly available datasets from blood (n = 3) and brain tissues (n = 3), separately. Firstly, we evaluated for each dataset and meta-analyzed the differential methylation profile between cases and controls. Secondly, we analyzed age acceleration, epigenetic drift and rare epigenetic variations.

**Results:**

We observed a significant epi-signature of ASD in blood but not in brain specimens. We did not observe significant age acceleration in ASD, while epigenetic drift was significantly higher compared to controls. We reported the presence of significant rare epigenetic variations in 41 genes, 35 of which were never associated with ASD. Almost all genes were involved in pathways linked to ASD etiopathogenesis (*i.e*., neuronal development, mitochondrial metabolism, lipid biosynthesis and antigen presentation).

**Conclusion:**

Our data support the hypothesis of the use of blood epi-signature as a potential tool for diagnosis and prognosis of ASD. The presence of an enhanced epigenetic drift, especially in brain, which is linked to cellular replication, may suggest that alteration in epigenetics may occur at a very early developmental stage (*i.e*., fetal) when neuronal replication is still high.

## 1 INTRODUCTION

Autism spectrum disorders (ASD) are a complex group of neurodevelopmental conditions characterized by impairment in social communication and interaction and by the presence of restricted patterns of behaviors and interests or sensory abnormalities. In the last decade, global interest for ASD has risen both in the public and among psychiatrists driven by a dramatic increase in prevalence (1 in 44 children) [1]. Despite the presence of high heritability and the identification of numerous genes which confer susceptibility to ASD, the precise definition of the clinical impact of genes involved in ASD is yet to be determined. Additionally, environmental factors impacting both the mother and the child could play a significant role in determining the course of neurodevelopment [2]. Until now, the search for causes and risk factors of ASD has faced several difficulties, among which the extreme phenotypic heterogeneity is probably the most relevant together with substantial genetic variability. Epigenetics represent a still underexplored field in ASD, but with the potential to maximize our knowledge. In general, epigenetics refers to the “heritable changes in gene expression without changing the underlying DNA sequence” [3]. Epigenetic regulation may act through different mechanisms, the most well-known being histone acetylation/deacetylation [4] and DNA methylation. DNA methylation generally blocks transcription at CpG islands near gene transcription start sites. More specifically, DNA methylation finely tunes levels of gene expression according to locus and developmental timing (*i.e*. specific genomic regions may be hypermethylated and therefore not expressed during childhood while being hypomethylated and expressed in adulthood) [5]. So far, the study of epigenetics in ASD has been primarily limited to the identification of differences between groups of patients and controls, while little attention has been devoted to other epigenetic aspects such as epigenetic age acceleration, epigenetic drift and the role of rare epigenetic variations (also known as epivariations). Additionally, literature studies, frequently underpowered, have reported inconsistent findings. The aim of the study was to conduct a more in-depth analysis of the role of epigenetics in autism, using only up-to-date high-throughput array raw data from public datasets. This would allow us to adopt an original multi-level approach starting with 1) a classic genome-wide epigenetic analysis (EWAS) and meta-analysis to improve the precision and accuracy of effect estimates and increase the statistical power to detect significant epigenetic effects, followed by 2) an analysis of more peculiar epigenetic issues such as biological aging, epigenetic drift and rare epigenetic variations.

## 2 MATERIALS AND METHODS

### 2.1 Selection of Datasets

We conducted a search using the term “autism” in Gene Expression Omnibus (GEO) data repository and EWAS Data Hub platform from inception to May 2022. To be included, studies should be case-control studies, reporting data using the Illumina HumanMethylation450 or Infinium MethylationEPIC platforms, in blood or in brain tissues. We included only studies with a sample size greater than 20. After the application of the previous criteria, 6 studies were eligible and included in the analyses [6-11]. Main information about selected datasets and sample size are summarized in Supplementary Table **S1**. The analysis was then performed on a total of 164 ASD cases and 102 controls considering blood samples and on a total of 49 ASD cases and 50 controls considering brain tissues.

### 2.2 Methylation Quality Control Data Preprocessing and Differential Analysis

The RnBeads package [12] was used for most of the analyses related to methylation data. Data from each dataset were analyzed separately using the same procedure (see Supplementary Methods). We assessed the presence of putative homozygous deletion regions by identifying clusters of probes with failed detection *p*-values as described by Barbosa *et al*. [13] and regions identified as putative homozygous deletion in each sample were then excluded from the list of DMR (Differentially Methylated Regions) calls. After the background correction, SWAN normalization was applied to each dataset. Immune cell composition, epigenetic age, age acceleration and GrimAge predictor of mortality values were inferred using the tool DNAmage calculator (https://horvath.genetics.ucla.edu/html/dnamage/). Based on data describing immune cell composition, Partial Least Squares (PLS) was used to extract principal components capable of summarizing the relationship between group (Y dependent variable) and cellular components (X vector of explanatory variables). Batch effect was identified with the exploratory analysis, checking for the presence of an association between the most important principal components and the sentrix-identifiers. The ComBat method [14] was used to adjust for batch effect where present. Differential Methylation Analyses were performed both at site level and at the genomic region level to find differences in methylation levels between cases and controls. This analysis was performed with hierarchical linear models implemented in the *limma* package [15]. Finally, differential methylation analyses were performed adjusting for potential confounders such as age, batch effect, PLS components obtained from immune cell composition and presence of a known genetic mutation such as presence of two type of genetic anomalies (16p11.2 deletions and CHD8 variants), which were recorded in the original article by Siu *et al*. [8]. The set of used covariates was evaluated for each study and is summarized in Supplementary Table **S1**. Both FWER (Family-wise Error Rate) and FDR (False Discovery Rate) approaches were adopted to address the problem of multiple tests. In particular, the procedure of Benjamin and Hochberg and Bonferroni correction were used. The R function *p.adjust* was used, putting as method “*BH*”, “*bonferroni*”. Age acceleration analysis The epigenetic aging measures for this study included the Horvath, Hannum, and GrimAge clocks and were obtained from the online DNA Methylation Age Calculator (https://dnamage.genetics.ucla.edu/) developed by Horvath [16] (Supplementary Methods).

### 2.3 Meta-Analysis

The meta-analysis was performed to combine both site and genomic-regions specific *p*-values obtained from differential methylation analyses. The software METAL, which is a specific tool to perform meta-analysis at genome-wide and epigenome-wide level, was used [17]. To obtain more consistent results, a meta-analytic approach was also applied to results from both the analysis of age acceleration and epigenetic drift. A mixed- effects model was applied, in which the hypothesis is that the observed differences could be due to differences between studies. The goal was to obtain a summary estimate for the effect size. This analysis was conducted through the R-supported “metaphor” package, which provided in addition to pooled estimates, statistics regarding heterogeneity such as the Q statistic and the I2 statistic [18]. Finally, the results were represented through a forest plot by using the Forest function of R's metafor package.

### 2.4 Stochastic Epigenetic Mutations (SEMs) Analysis and Epivariation Analysis

To identify Stochastic Epigenetic Mutations (SEMs), a method already described by Gentilini *et al*. was used [19-22]. SEMs were identified as aberrant beta-values (defined as extreme outliers) falling outside a reference methylation range obtained by the methylation profiles of a reference population and calculated as follows: upper value = Q3 + (k*IQR); lower value = Q1-(k*IQR); where Q1 is the first quartile, Q3 the third quartile, IQR (Interquartile range) = Q3-Q1 and k = 3. For each case, extreme outlier values of single methylation profiles were annotated and classified as hyper-methylated or hypo-methylated to controls' relative probe median values. The burden of SEMs was expressed in logarithmic scale and compared between cases and controls through a logistic regression model and taking into account the same set of covariates used in the EWAS step; differences were expressed as odds ratios. To detect the regions enriched in SEMs or epigenetic variations, defined from now onwards as “Epivariation”, we adopted the method previously described and validated by Gentilini *et al*. [21-23]. An over-representation analysis of all identified SEMs was conducted by using a sliding window algorithm based on a cumulative hypergeometric distribution which tests the significant enrichment of SEMs in a window of a predefined size (*e.g*., 11 CpG sites) that slips (by single sites) on the annotated genome generating a window-associated *p*-value. The procedure is repeated in the adjacent windows generating a list of SEM-enriched regions. The R package adopted to calculate SEMs is published at DOI: 10.5281/zenodo.3813234.

### 2.5 GeneSet Enrichment Analysis, Comparative Toxicogenomics and Gene Prioritization Analysis

GeneSet Enrichment Analysis was performed using the WEB-based GEne SeT AnaLysis Toolkit (WebGestalt) [24]. Comparative Toxicogenomics Analysis was performed using the Comparative Toxicogenomics Database web tool, a public resource that integrates chemical and genomic data [25] (CTD; http://ctdbase.org). The Prioritization analysis was performed following a heuristic approach to highlight the most interesting genes associated with epivariations. The aim of this analysis was to obtain a list of genes, absent in controls and shared among cases. Moreover, we gave greater importance to genes expressed in the brain or playing a role in regulating behavior. A schematic representation of the adopted strategy is described in Supplementary Fig. (**S1**) and Supplementary Methods.

## 3 RESULTS

### 3.1 Exploratory and Differential Methylation Analysis

No macroscopic methylation differences emerged between cases and controls, neither in blood nor brain tissues. Results of exploratory analysis are shown in Supplementary Fig. (**S2**). Differential methylation analysis in blood and brain tissues was performed at probe level and results are reported in Fig. (**1**). Considering the blood tissues, only data from the article by Siu *et al*. [8] showed 274 probes differently methylated while datasets from Homs *et al*. [6] and Kimura *et al*. [7] did not show genome wide significant results. The results obtained from brain tissue studies did not reveal any significant probes after Bonferroni correction.

### 3.2 Meta-analysis of Differential Methylation Results and Gene Set Enrichment Analysis

Considering the blood tissues, after FDR multiple testing correction, 3623 probes resulted differently methylated in cases. Results of the meta-analysis are reported in Supplementary Table **S2**. Based on the resulting probes, a gene set enrichment analysis (GSEA) was performed focusing on gene ontology, KEGG pathways and disease levels to better understand the biological function of the observed epigenetic changes. The GSEA identified several enriched gene ontology Biological Processes, among which the most interesting were dendrite development and synapse organization. Moreover, the enrichment analysis performed at KEGG pathway level showed a significant enrichment in Axon guidance pathway. Finally, the GSEA analysis identified a significant enrichment in genes involved in pathological conditions characterized by the presence of intellectual disability. The complete list of significant results obtained from GSEA analysis is shown in Supplementary Table **S3**. Conversely, considering the brain tissues, the meta-analysis did not show statistically significant results (Supplementary Table **S4**).

### 3.3 Comparative Toxicogenomics Database Analysis

We search the Comparative Toxicogenomics Database for each gene of the episignature to obtain a list of associated chemical compounds. Then, the list of chemicals was sorted according to the number of corresponding genes to identify the strongest associations. The first five most interesting results were: Valproic Acid, benzo-(a)-pyrene, bisphenol A, tobacco smoke pollution, Aflatoxin B. The complete list is reported in Supplementary Table **S5**.

### 3.4 Age Acceleration

**Fig. (2):**
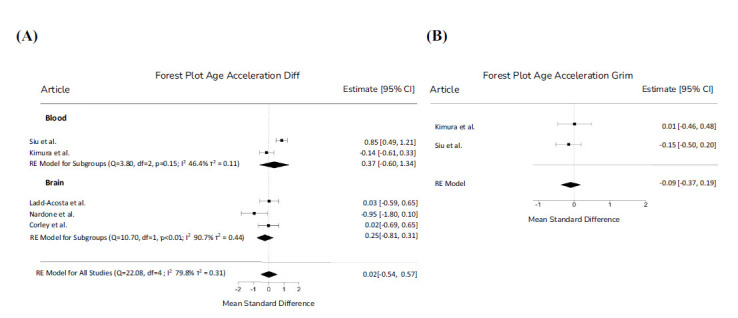
(**A**) Forest Plot for Age Acceleration Diff; (**B**) Forest Plot for age Acceleration Grim Forest plot of the effect size or standardized mean difference and 95% confidence interval (CI) of the effect of case control status on Acceleration Diff (panel **A**) and Acceleration Grim (panel **B**). The dashed vertical line represents a mean difference of 0 or no effect. Squares to the left of the line represent a decrease in Age Acceleration Diff or Age Acceleration Grim while squares to the right of the line indicate an increase. Each square represents the mean effect size for that study and reflects the relative weighting of the study to the overall effect size estimate. The larger the box, the greater the study contribution to the overall estimate.

Epigenetic age was evaluated in all subjects, whose chronological age was available. Age acceleration was calculated both in brain and blood specimens and, subsequently, the calculated age acceleration estimates were combined through a meta-analysis (Fig. **2A**). No significant difference in age acceleration between cases and controls in blood and brain tissues was observed. Furthermore, only in blood samples, the GrimAge predictor of mortality was evaluated and no significant difference between cases and controls emerged from the meta-analysis (Fig. **2B**).

### 3.5 Epigenetic Drift

There was a significantly increased epigenetic drift in ASD subjects, both in blood and brain tissues. Considering blood tissue, the OR for SEMs was 1.68 (CI = 1.07-2.64), while in brain tissue, the OR for SEMs was 2.39 (CI = 1.31-4.37). Results of the meta-analysis considering total SEMs are shown in Fig. (**3**).

### 3.6 Epivariation Analysis

**Fig. (4):**
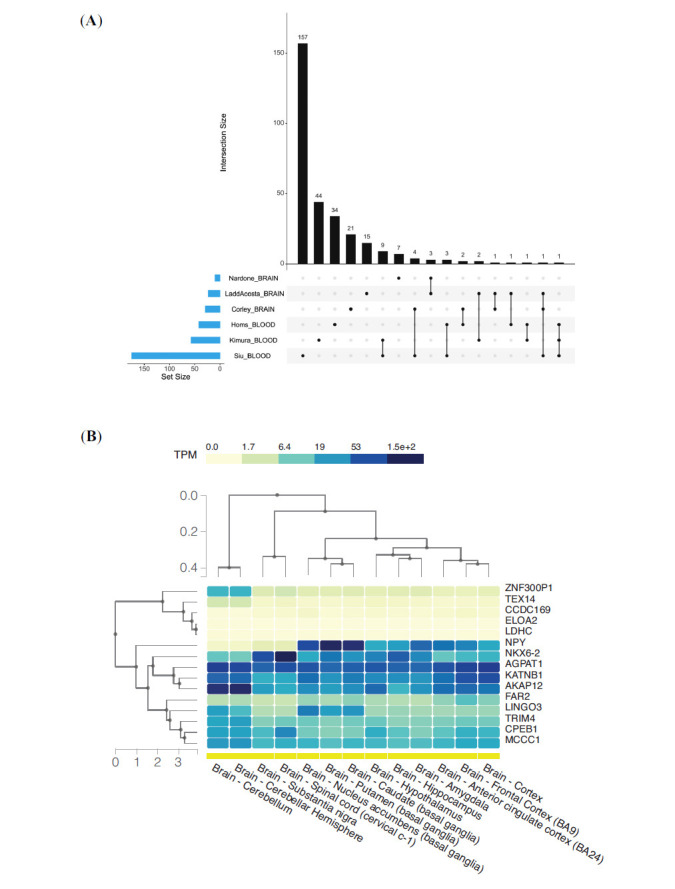
(**A**) Upset plot of all gene overlaps among studies, (**B**) Genes found in more than three cases annotated with their average level of gene expression in 12 different brain regions generated by the GTEx project.

Based on SEMs it was also possible to significantly identify SEM-enriched regions that are also known as epivariations. For each subject, coordinates of genomic epivariations (SEM-enriched regions) were reported and annotated to obtain the list of genes involved. The list of genes with the description of tissue, methylation status (up or down methylated), sample group and number of subjects is reported in Supplementary Table **S6**. Finally, considering both brain and blood results, the list of genes found only in cases was evaluated. Case unique genes were then compared among studies to find suggestive overlaps. The upset plot in Fig. (**4A**) describes all the overlaps among studies. In blood, 13 genes (KATNB1, TRIM4, ELOA2, ASTE1, TEX14, EFCAB10, LDHC, TRIM39-RPP21, OR4D9, ZNF300P1, OSBP, NPY, HLA-E) were shared by at least two studies while one gene (KATNB1) emerged from all the studies. Results from the brain identified five genes shared by at least two studies (SFMBT1, NKX6-2, CFAP46, GRIK2, LINGO3). A complete list of 306 genes was then obtained considering genomic epivariations found either in brain or blood tissues. Among these genes, nine genes (CCDC169-SOHLH2, FDFT1, C6orf48, WNK4, AKAP12, PLD6, CCDC169, FAR2, LINGO3) were shared by both tissues while, if we consider the number of cases sharing the same epivariation, 17 genes were shared by more than three unrelated cases. Using gene expression data from the GTEx project (https://www.gtexportal.org/home/), the set of 17 genes found in more than three cases was annotated with their mean gene expression level in 12 different brain regions (Fig. **4B**). The analysis highlighted a cluster of 10 genes showing high expression levels in brain (NPY, NKX6-2, AGPAT1, KATNB1, AKAP12, FAR2, LINGO3, TRIM4, CPEB1, MCCC1).

### 3.7 Epivariations Prioritization Analysis

**Fig. (5):**
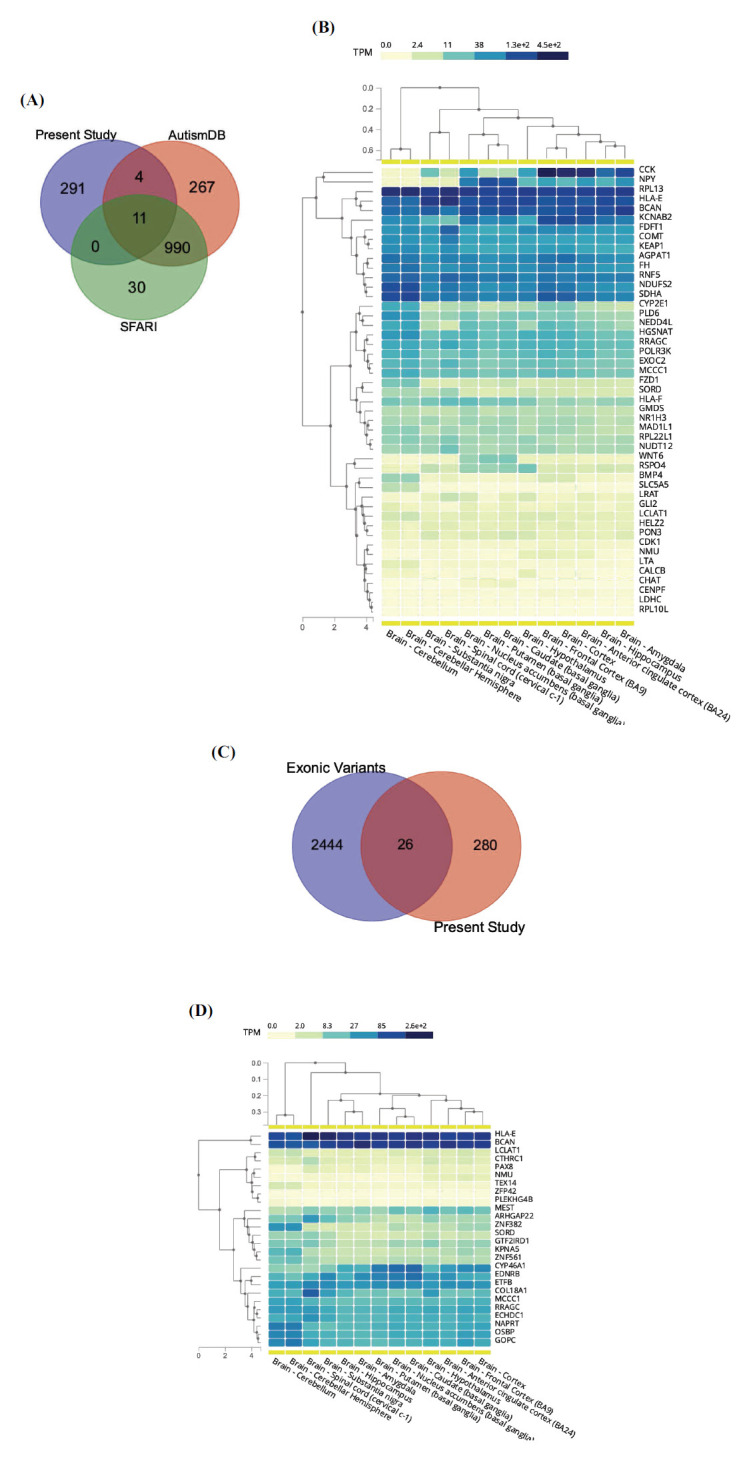
(**A**) Venn diagram represents the intersection between our epivariation associated gene list, Sfari Genes and autism Database Genes; (**B**) Annotation of top 50 highest prioritization scores genes obtained from the prioritization analysis with phenolyzer using the HPO term “Behavior”. The image shows their average level of gene expression in 12 different brain regions generated by the GTEx project; (**C**) Venn diagram represents the intersection between our epivariation associated gene list and a list of genes obtained from Autism Sequencing Consortium exome analysis characterized by presence of rare missense or stop codon variants found only in ASD subject and never in controls. (**D**) Genes obtained from the intersection are annotated with their average level of gene expression in 12 different brain regions generated by the GTEx project.

The complete list of epivariations was intersected with genes known to be associated with ASD to eventually identify new gene targets. In total, 15 genes overlapped with already known ASD genes (Fig. **5A**). We focused on the 291 genes not yet reported as associated with ASD, using a prioritization strategy considering “behavior” as a unique HPO term to obtain a list of top 50 genes showing highest prioritization scores. These genes were finally annotated with their mean gene expression level in 12 different brain regions generated by the GTEx project. Results are shown in Fig. (**5B**). The list of 291 genes was also compared with a list of genes obtained from Autism Sequencing Consortium exome analysis characterized by presence of rare missense or stop codon variants found only in ASD. We were able to identify 25 genes (Fig. **5C**) that were affected by rare variants in exome experiments. These genes were finally annotated with their mean gene expression level in 12 different brain regions generated by the GTEx project. Results are shown in Fig. (**5D**). The prioritization procedure ended with the identification of 41 priority genes, 35 of which have never been previously described. Supplementary Table **S7** shows the complete list of genes with a brief description.

## 4 DISCUSSION

The present study investigated the role of epigenetics in ASD, focusing on DNA methylation. Based on a systematic analysis of public data repositories, six datasets (three from blood and three from brain samples) containing genome wide methylation data were identified and re-analyzed. We conducted a multilevel epigenome-wide analysis: in the first step, we evaluated epigenetic differences associated with ASD, using the usual EWAS approach; secondly, we incorporated in the analysis more peculiar epigenetic issues such as biological aging, epigenetic drift and rare epigenomic variations. The first analysis found an epigenetic signature significantly associated with ASD at genome wide level in blood, but not in brain tissues. Additionally, the gene set enrichment analysis confirmed that genes observed in the epigenetic signature were known to be involved in gene ontology functions, pathways and phenotypic traits strongly associated with ASD, further strengthening the potential usefulness of the epigenetic signature. Specific methylation patterns for ASD have been inconsistently reported in literature in different tissues [26] and epi-signatures associated to a higher risk for ASD have been observed also in maternal and fetal blood [27]. Our results are also at least in part in line with findings from a large rigorous case-control meta-analytic study from Andrews *et al*. [28]: they observed an epi-signature in blood samples composed by only 48 CpG islands with low significance; they attributed this limited result to the type of sample used for the analysis; in fact almost half of the their sample was composed by siblings of probands, which share the same environmental background; this could have impacted epigenetic modification which could be similar between dyads [29]. Our study included only unrelated subjects, enhancing environmental heterogeneity as in the general population. Additionally, Andrews *et al*. [28] used the surrogate variables analysis to account for potential hidden biological and artificial factors. Although this method is considered robust, the used algorithms may reduce intragroup biological heterogeneity and thus specific epigenomic signatures. This could be relevant in case-siblings studies in which exposure factors are shared between cases and controls. In accordance with our hypothesis, Andrews *et al*. [28] also suggested that blood epi-signatures can be reflective of epi-signatures in other tissues and, given the feasible collection of blood specimen *versus* brain specimen, the utility of blood-based epigenetic in ASD is still worthy of additional investigation for early diagnosis [30] as well as accurate prediction of prognosis [31]. Our null findings in brain samples could be related to the low sample size as well as to the type of cerebral tissue (*i.e*., frontal cortex). As the epigenetic profile could change after exposure to several types of environmental factors, we analyzed the observed epi-signature for potential association with chemically relevant compounds. Our results showed several chemical compounds already known or suspected to be involved in the pathogenesis of ASD. For instance, valproic acid was the compound associated with the highest number of genes of the epi-signature. This is in line with results from literature showing that children prenatally exposed to valproic acid have an increased chance of being diagnosed with ASD [32]. Additionally, we observed a potential association with other chemical compounds such as benzo-(a)-pyrene, bisphenol A and tobacco smoke pollution which have been already identified as risk factors for ASD [33]. Our results also showed an association with Mycotoxins, in particular Aflatoxin B, which have a more controversial role in ASD with several studies reporting conflicting findings [34]. Our data support the hypothesis of a more pronounced role of Aflatoxin B in ASD. It is important to underline that using epi-signature as a tool to investigate environmental factors potentially involved in different psychiatric conditions represents an innovative approach in psychiatry, which seems at least in part validated by the large amount of literature data coming from observational and exposure studies in ASD. The second step of our analysis evaluated epigenetic aging and the age acceleration. Studies evaluating age acceleration in ASD have so far obtained conflicting results [35, 36]. Our meta-analysis of the 6 datasets supported the absence of significant accelerated aging in ASD subjects both in brain and blood specimens; additionally, we did not observe a significant risk of mortality in blood samples. This result could be of importance as it might shed more light on the prognosis of ASD subjects, and cautiously suggest to the scientific community to reconsider the biological causes of the reported increased mortality in ASD [37, 38]. In fact, ASD mortality is due mostly to unnatural causes (such as accidents) or natural causes generally not associated with life-style habits (*i.e*. epilepsy, polypharmacy, *etc*), while the GrimAge clock may be a more reliable proxy of all-cause mortality linked with unhealthy life-style habits (tobacco smoking, cardiovascular deaths) [39]. A completely different picture emerged when we considered the epigenetic drift. Our study showed for the first time that stochastic epimutation load, considered a valid indicator of epigenetic drift, was significantly increased in ASD subjects both in blood and brain tissues. Epigenetic drift can be defined as the accumulation of mistakes in maintaining normal epigenetic patterns leading to an increase in variability of epigenetic marks throughout the individual’s life course. This process usually results in a gradual impairment of the body’s homeostatic mechanisms and contributes to impaired cellular and molecular functions and to the decline in phenotypic plasticity at cellular and molecular levels [40]. The increased epigenetic drift in ASD, especially in the brain, and the potential detrimental effect on cellular and tissue plasticity is by itself able to explain some characteristics of ASD, for instance the presence of an altered microanatomy of the brain tissue [41]. Moreover, it is well-known that young cells have relatively uniform epigenomes but, during cell replication, stochastic errors in the maintenance of DNA methylation occur, causing epigenetic mosaicism [42]. In line with this hypothesis, recent evidence showed increased genetic mosaicism in the cerebral cortex of ASD subjects [43]. Of note, as neuronal plasticity and brain cellular turn-over dramatically decreases after birth and stochastic errors relate to cell replication, it is possible to hypothesize that the genetic mosaicism [43] and the observed age-adjusted increased epigenetic drift in ASD may originate during an early stage of development. Consequently, our results may support the fetal or early neonatal age of onset of ASD. Finally, we have studied the presence and relevance of rare epivariations. There is growing evidence that rare epivariations are associated with several human diseases [23] and play a key pathogenetic role in a subgroup of congenital and neurodevelopmental disorders through the disruption of dosage-sensitive genes [44]. We applied a validated approach able to identify rare epivariations and a prioritization strategy in order to highlight genes potentially relevant for ASD. The analysis led to a small set of 41 genes, 35 of which have never been described before in the field of ASD but are extremely interesting considering their function, their role in the regulation of behavior and their level of expression in the brain. Most of these unknown identified genes are related to RNA production and transcription, oxidative metabolism in mitochondria, lipid biosynthesis and dendritic, axonal, myelinic and synaptic development (Supplementary Table **S7**). All these mechanisms and pathways have been already identified in ASD as impaired [45-49]. Within this list, the NPY gene is a pleiotropic central nervous system gene, particularly interesting for its potential therapeutic perspectives. Despite not being associated with ASD so far, it is involved in the regulation of emotional as well as anxiety-related behaviors and has been associated with depression [50]. In mice models, activation of neuropeptide Y neurons has been linked to the presence of stereotypic ASD-like behaviors [51]. The neuropeptide Y system is also emerging as a promising therapeutic target for neuropsychiatric disorders by intranasal delivery to the brain [52]. Both animal and human studies have reported an anxiolytic effect after NPY administration [53, 54]. Moreover, chemical compounds with selective agonism on Y1 receptor mediates the anxiolytic and antidepressant effects of NPY [55, 56] as well as, coupled with agonism at the Y2R, regulates fear conditioning [57]. As all these mechanisms are relevant in ASD [58, 59], NPY could be a potential promising target for future studies. The present study has strengths and limitations. It is the first study evaluating epigenetics using a wider perspective: we moved from a mere comparison of cases and controls to include different epigenetic features like age acceleration and epigenetic drift and rare epivariations. Thanks to an approach based on re-analysis of data and subsequent meta-analysis, we were able to reduce variability among analyses and to obtain a significant robust epi-signature for ASD. For the first time, we provided evidence of altered epigenetic drift in ASD, thus enabling future lines of research in this area. Finally, we extended the knowledge on rare epivariations in ASD, which were previously observed only in a small cohort [44]. Study limitations must be considered to prevent overinterpretation of our findings. Firstly, we selected only a small subset of publicly available data: this was done to obtain more robust findings and to avoid all conditions known to lower study power and increased variability (*i.e*. small sample size datasets and low-throughput BeadChip) [60]. Another pitfall is related to the low number of phenotypic data available which limited the possibility of more in-depth analyses.

## CONCLUSION

ASD is associated with an epi-signature in blood specimens, which could be the potential feasible target of future research. Additionally, our results on increased epigenetic drift, especially in brain tissues, shed more light on potential precocious etiopathogenetic processes and pave the way for future research focused on prenatal exposures leading to brain mosaicism. Finally, rare epivariations may represent an underexplored diagnostic field, which could allow clinicians to better tailoring heterogeneity of ASD; moreover, they may also provide insight for therapeutic targeting of new molecules (*i.e*. NPY) or pathways in ASD.

## Figures and Tables

**Fig. (1):**
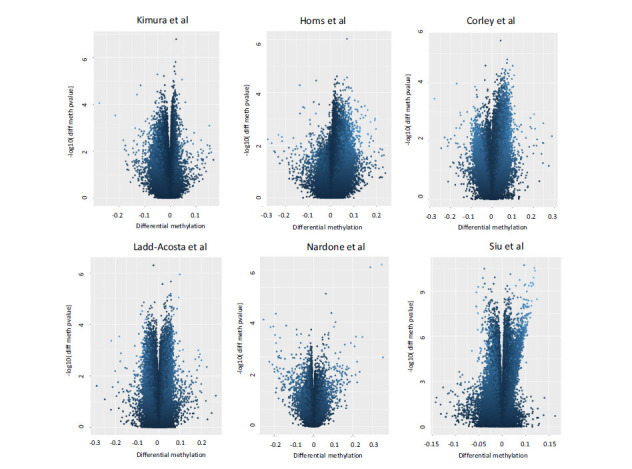
Volcano plot of differences in DNA methylation between the ASD and Controls. Each panel describes results obtained from a single study. Each point represents a CpG site with mean differences in DNA methylation between groups on the x-axis and−log10 of the uncorrected *P* value from empirical bayes moderated t-test on the y-axis. Negative methylation differences indicate hypomethylation and positive differences indicate hypermethylation.

**Fig. (3):**
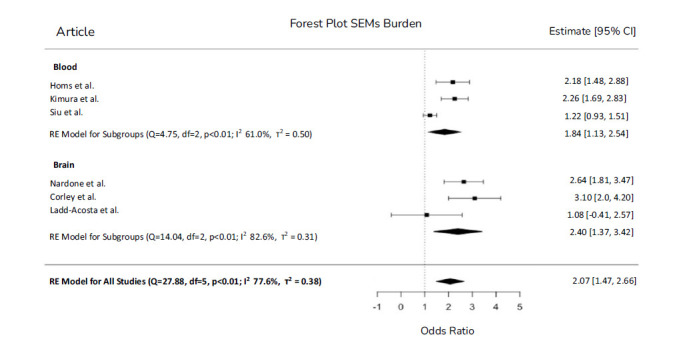
Forest Plot for Epigenetic drift. Forest plot describes the effect size expressed as Odds Ratio and 95% confidence interval (CI) obtained from a multiple logistic regression model evaluating the effect of Epigenetic drift on case control status. The dashed vertical line represents an OR of 1 or no effect. Squares to the left of the line represent a decrease in OR while squares to the right of the line indicate an increase. Each square represents the mean effect size for that study and reflects the relative weighting of the study to the overall effect size estimate. The larger the box, the greater the study contribution to the overall estimate.

## Data Availability

Not applicable.

## LIST OF ABBREVIATIONS

ASD: Autism Spectrum Disorders
DMR: Differentially Methylated Regions
EWAS: Genome-wide Epigenetic Analysis
FDR: False Discovery Rate
FWER: Family-wise Error Rate
GEO: Gene Expression Omnibus
GSEA: Gene Set Enrichment Analysis
IQR: Interquartile Range
PLS: Partial Least Squares
SEMs: Stochastic Epigenetic Mutations
