# Supplementary material for: Epigenetics of Autism Spectrum Disorders: A Multi-level Analysis Combining Epi-signature, Age Acceleration, Epigenetic Drift and Rare Epivariations Using Public Datasets
Source: Curr Neuropharmacol. 2023 Sep 1;21(11):2362–73. doi: 10.2174/1570159X21666230725142338 (PMC10556384; doi:10.2174/1570159X21666230725142338)
Supplement: Supplementary file 1 [file CN-21-2362_SD1.pdf]

Supplementary Material

Epigenetics of Autism Spectrum Disorders: A Multi-level Analysis Combining Epi-signature, Age Acceleration, Epigenetic Drift and Rare Epivariations Using Public Datasets

Gentilini Davide[1,2,*], Cavagnola Rebecca[1], Possenti Irene[3], Calzari Luciano[2], Ranucci Francesco[1], Nola Marta[1], Olivola Miriam[1], Brondino Natascia[1,#] and Politi Pierluigi[1,#]

[1]*Department of Brain and Behavioral Sciences, University of Pavia, 27100 Pavia, Italy;* [2]*Bioinformatics and Statistical Genomics Unit, IRCCS Istituto Auxologico Italiano, 20090 Milan, Italy;* [3]*Department of Statistical Sciences Paolo Fortunati, University of Bologna, Bologna, Italy*

1875-6190/23 $65.00+.00 **© 2023 Bentham Science Publishers**

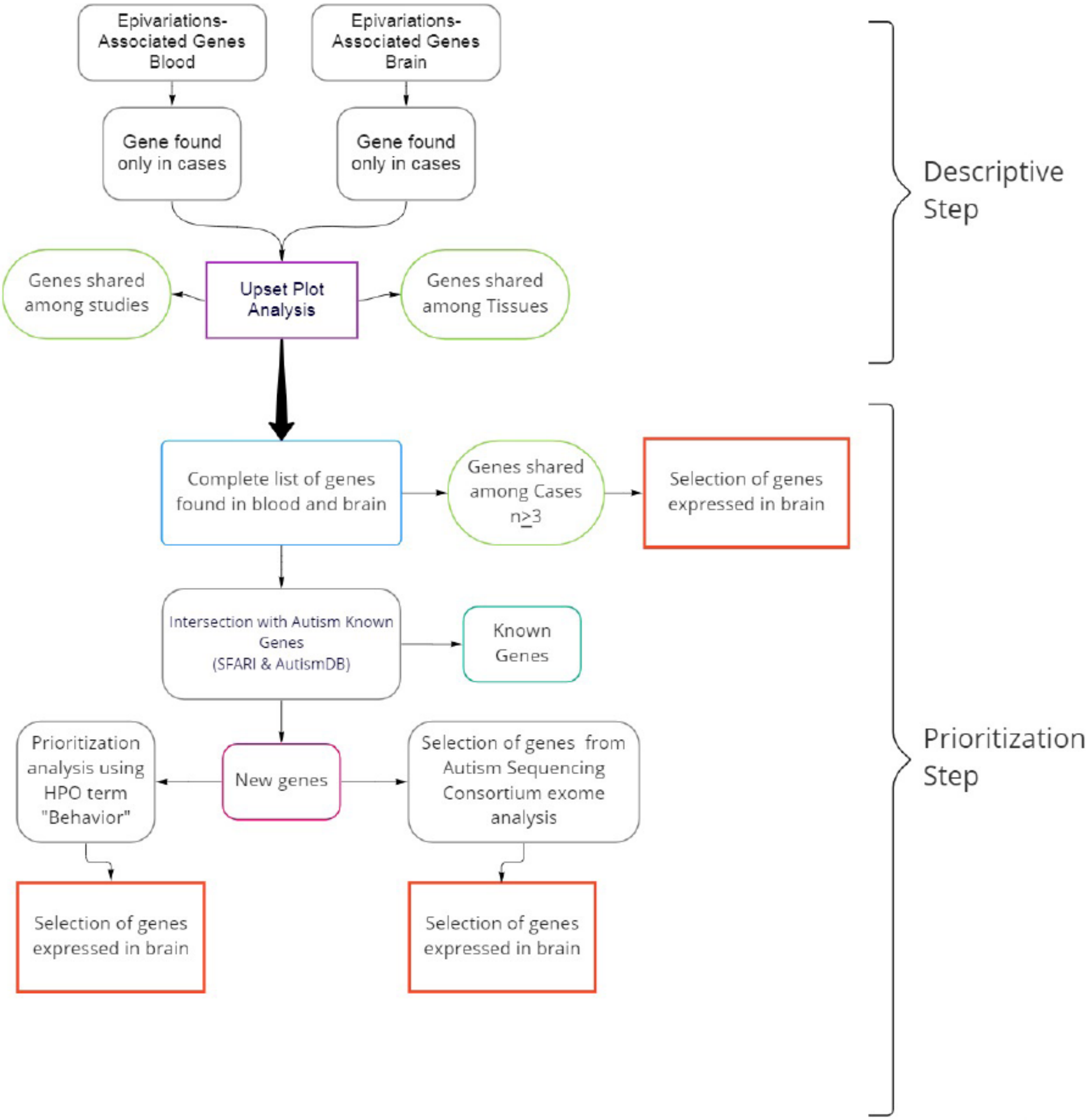

Fig. (S1). Schematic representation of strategy adopted for Prioritization analysis.

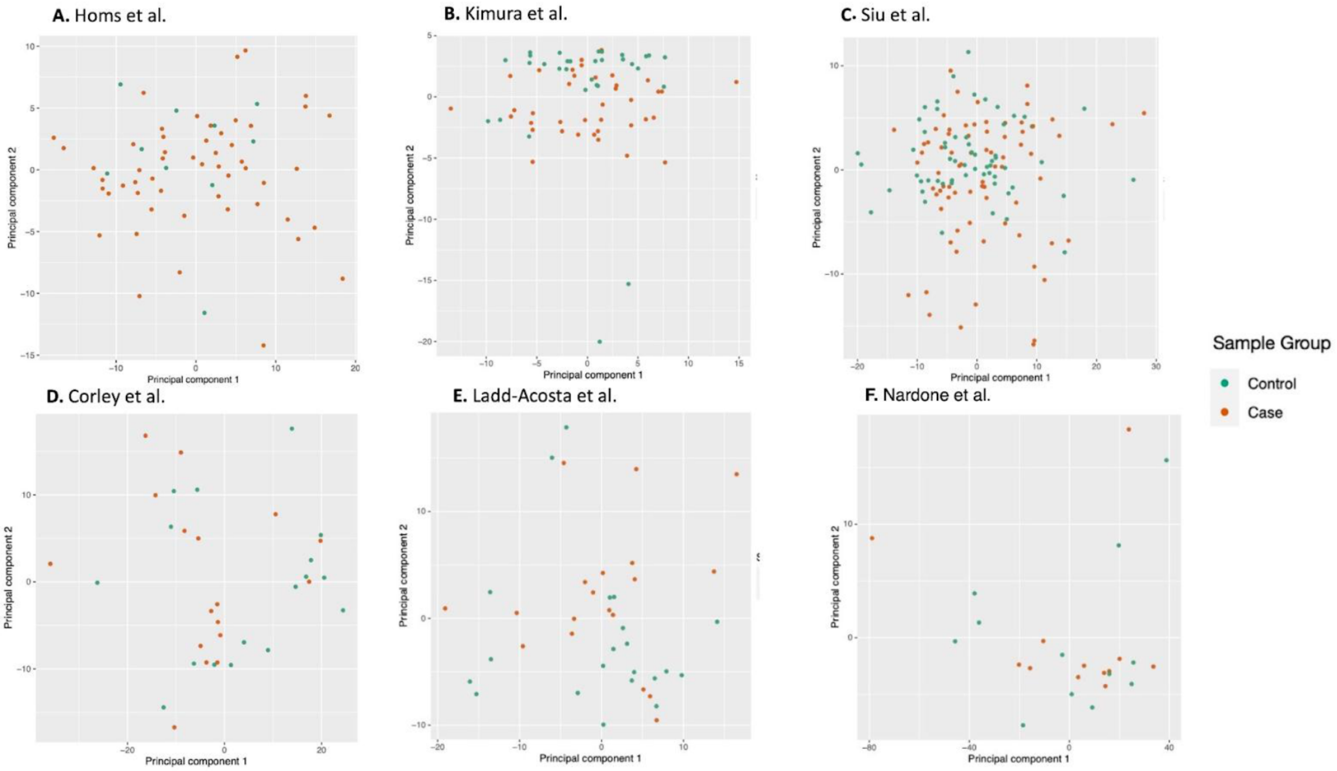

Fig. (S2). Exploratory analysis. Each *Scatter plot* describes Genome wide methylatio profiles by using the *first two principal components obtained from methylation l evels of probes.*

Supplementary Table S1: Main information about selected datasets, sample size and Covariates used in the differential analyses.

First Author	GSE	Publication Year	Country	# Cases	# Controls	Tissue	Array Type	Set of Covariates used in the Differential Analysis
**Homs *et al.***	GSE83424	2016	Spain	53	10	Blood	Illumina 450K	Age
**Kimura *et al.***	GSE109905	2019	Japan	38	31	Blood	Illumina 450K	Age, Gender, Cellular composition (PC1)
**Siu *et al.***	GSE113967	2019	Canada	73	61	Blood	Illumina 450K	Age, Gender, Type of genetic Mutation
**Ladd-Acosta *et al.***	GSE53162	2014	USA	19	21	Brain	Illumina 450K	Age, Gender
**Corley *et al.***	GSE131706	2019	USA	17	17	Brain	Illumina 450K	Age
**Nardone *et al.***	GSE53924	2014	Israel	13	12	Brain	Illumina 450K	Age, Gender

Supplementary Table S3

**Enrichment Categories:** Geneontology_Biological_Process				
**Gene Set**	**Description**	**Enrichment Ratio**	**P Value**	**FDR**
GO:0050808	synapse organization	1.5624	7.99E-05	0.009707
GO:0016358	dendrite development	1.8182	3.07E-05	0.006532
GO:0042176	regulation of protein catabolic process	1.6944	3.09E-06	0.001777
GO:0043087	regulation of GTPase activity	1.5975	4.18E-06	0.001777
GO:0040013	negative regulation of locomotion	1.6728	2.87E-05	0.006532
GO:0022604	regulation of cell morphogenesis	1.5045	7.42E-05	0.009707
GO:0051056	regulation of small GTPase mediated signal transduction	1.6125	7.77E-05	0.009707
GO:0051271	negative regulation of cellular component movement	1.6325	0.000104	0.011038
GO:0006352	DNA-templated transcription, initiation	1.7237	0.000122	0.011516
GO:0040029	regulation of gene expression, epigenetic	1.6747	0.000138	0.01171
**Enrichment Categories:** pathway_KEGG				
**Gene Set**	**Description**	**Enrichment Ratio**	**P Value**	**FDR**
hsa04360	Axon guidance	1.8685	8.54E-05	0.027851
**Enrichment Categories:** disease_Disgenet				
**Gene Set**	**Description**	**Enrichment Ratio**	**P Value**	**FDR**
C0036341	Schizophrenia	1.4171	9.45E-07	0.00347
C3714756	Intellectual Disability	1.4198	3.78E-05	0.035235
C0234162	Cerebellar Dysmetria	2.7643	6.54E-05	0.035235
C0025362	Mental Retardation	1.4311	8.37E-05	0.035235
C0423903	Low intelligence	1.4311	8.37E-05	0.035235
C0917816	Mental deficiency	1.4311	8.37E-05	0.035235
C1843367	Poor school performance	1.4311	8.37E-05	0.035235
C4020876	Dull intelligence	1.4311	8.37E-05	0.035235
C4021371	Absent mandible	7.5883	8.63E-05	0.035235
C1836542	Depressed nasal bridge	1.8119	0.000152	0.04646

Supplementary Table S6

Gene Symbol	Dataset	Author	Up/Down Methylated	#Cases	# Controls
BTNL3	Autism_GSE53162_Brain	Ladd-Acosta *et al.*	down	0	1
BTNL8	Autism_GSE53162_Brain	Ladd-Acosta *et al.*	down	1	1
BTNL8	Autism_GSE53162_Brain	Ladd-Acosta *et al.*	down	1	1
DUSP22	Autism_GSE53162_Brain	Ladd-Acosta *et al.*	down	1	1
HLA-DOA	Autism_GSE53162_Brain	Ladd-Acosta *et al.*	down	1	0
HLA-DPB2	Autism_GSE53162_Brain	Ladd-Acosta *et al.*	down	1	0
PRRT1	Autism_GSE53162_Brain	Ladd-Acosta *et al.*	down	1	0
SFMBT1	Autism_GSE53162_Brain	Ladd-Acosta *et al.*	down	1	0
TCERG1L	Autism_GSE53162_Brain	Ladd-Acosta *et al.*	down	1	0
WDR88	Autism_GSE53162_Brain	Ladd-Acosta *et al.*	down	0	1
ACTR3C	Autism_GSE53162_Brain	Ladd-Acosta *et al.*	up	0	1
AKAP12	Autism_GSE53162_Brain	Ladd-Acosta *et al.*	up	1	0
C6orf48	Autism_GSE53162_Brain	Ladd-Acosta *et al.*	up	1	0
CCDC140	Autism_GSE53162_Brain	Ladd-Acosta *et al.*	up	1	1
CENPF	Autism_GSE53162_Brain	Ladd-Acosta *et al.*	up	1	0
CHAT	Autism_GSE53162_Brain	Ladd-Acosta *et al.*	up	1	0
DBX1	Autism_GSE53162_Brain	Ladd-Acosta *et al.*	up	1	1
DHX40	Autism_GSE53162_Brain	Ladd-Acosta *et al.*	up	1	0
DUSP1	Autism_GSE53162_Brain	Ladd-Acosta *et al.*	up	1	1
EN1	Autism_GSE53162_Brain	Ladd-Acosta *et al.*	up	1	0
FABP5P3	Autism_GSE53162_Brain	Ladd-Acosta *et al.*	up	0	1
FAM24B-CUZD1	Autism_GSE53162_Brain	Ladd-Acosta *et al.*	up	1	0
GRIK2	Autism_GSE53162_Brain	Ladd-Acosta *et al.*	up	1	0
GSDMD	Autism_GSE53162_Brain	Ladd-Acosta *et al.*	up	0	1
GSTT1	Autism_GSE53162_Brain	Ladd-Acosta *et al.*	up	1	1
GSTT1	Autism_GSE53162_Brain	Ladd-Acosta *et al.*	up	0	1
HKR1	Autism_GSE53162_Brain	Ladd-Acosta *et al.*	up	1	0
INTU	Autism_GSE53162_Brain	Ladd-Acosta *et al.*	up	0	1
ISOC2	Autism_GSE53162_Brain	Ladd-Acosta *et al.*	up	1	1
KMT2C	Autism_GSE53162_Brain	Ladd-Acosta *et al.*	up	0	1
L3MBTL1	Autism_GSE53162_Brain	Ladd-Acosta *et al.*	up	0	1
LINGO3	Autism_GSE53162_Brain	Ladd-Acosta *et al.*	up	1	0
MCCC1	Autism_GSE53162_Brain	Ladd-Acosta *et al.*	up	0	1
NAPRT	Autism_GSE53162_Brain	Ladd-Acosta *et al.*	up	1	0
NBPF25P	Autism_GSE53162_Brain	Ladd-Acosta *et al.*	up	0	1
NKX6-2	Autism_GSE53162_Brain	Ladd-Acosta *et al.*	up	1	0
NKX6-2	Autism_GSE53162_Brain	Ladd-Acosta *et al.*	up	1	0
NONE	Autism_GSE53162_Brain	Ladd-Acosta *et al.*	up	1	0
OLIG3	Autism_GSE53162_Brain	Ladd-Acosta *et al.*	up	1	1
SDHAP3	Autism_GSE53162_Brain	Ladd-Acosta *et al.*	up	1	1
SLC5A5	Autism_GSE53162_Brain	Ladd-Acosta *et al.*	up	1	0
SPAG1	Autism_GSE53162_Brain	Ladd-Acosta *et al.*	up	0	1
SPAG17	Autism_GSE53162_Brain	Ladd-Acosta *et al.*	up	1	0
TLX3	Autism_GSE53162_Brain	Ladd-Acosta *et al.*	up	1	1
TLX3	Autism_GSE53162_Brain	Ladd-Acosta *et al.*	up	1	1
TPTEP1	Autism_GSE53162_Brain	Ladd-Acosta *et al.*	up	0	1

Gene Symbol	Dataset	Author	Up/Down Methylated	#Cases	# Controls
TRMT10C	Autism_GSE53162_Brain	Ladd-Acosta *et al.*	up	0	1
TUBA1C	Autism_GSE53162_Brain	Ladd-Acosta *et al.*	up	0	1
URAD	Autism_GSE53162_Brain	Ladd-Acosta *et al.*	up	1	1
VSX1	Autism_GSE53162_Brain	Ladd-Acosta *et al.*	up	1	1
ZNF681	Autism_GSE53162_Brain	Ladd-Acosta *et al.*	up	0	1
ZNF732	Autism_GSE53162_Brain	Ladd-Acosta *et al.*	up	0	1
ACY3	Autism-GSE83424_Blood	Homs *et al.*	down	1	1
ADCY10P1	Autism-GSE83424_Blood	Homs *et al.*	down	0	1
ALOX12P2	Autism-GSE83424_Blood	Homs *et al.*	down	1	0
CASZ1	Autism-GSE83424_Blood	Homs *et al.*	down	0	1
CNTNAP1	Autism-GSE83424_Blood	Homs *et al.*	down	0	1
ELOA2	Autism-GSE83424_Blood	Homs *et al.*	down	1	0
EXOC2	Autism-GSE83424_Blood	Homs *et al.*	down	0	1
GFI1	Autism-GSE83424_Blood	Homs *et al.*	down	0	1
HOXA3	Autism-GSE83424_Blood	Homs *et al.*	down	1	0
HUS1B	Autism-GSE83424_Blood	Homs *et al.*	down	0	1
LDHC	Autism-GSE83424_Blood	Homs *et al.*	down	0	1
LOC102724511	Autism-GSE83424_Blood	Homs *et al.*	down	0	1
MIMT1	Autism-GSE83424_Blood	Homs *et al.*	down	1	0
MIMT1	Autism-GSE83424_Blood	Homs *et al.*	down	1	0
MPIG6B	Autism-GSE83424_Blood	Homs *et al.*	down	0	1
NFYA	Autism-GSE83424_Blood	Homs *et al.*	down	1	1
PEG3	Autism-GSE83424_Blood	Homs *et al.*	down	1	0
RPH3AL	Autism-GSE83424_Blood	Homs *et al.*	down	0	1
TRIM31-AS1	Autism-GSE83424_Blood	Homs *et al.*	down	0	1
ZFP57	Autism-GSE83424_Blood	Homs *et al.*	down	1	0
ACTR3C	Autism-GSE83424_Blood	Homs *et al.*	up	0	1
AKAP12	Autism-GSE83424_Blood	Homs *et al.*	up	1	0
ALKBH3	Autism-GSE83424_Blood	Homs *et al.*	up	1	1
ASTE1	Autism-GSE83424_Blood	Homs *et al.*	up	1	0
B4GALNT4	Autism-GSE83424_Blood	Homs *et al.*	up	1	0
BBS10	Autism-GSE83424_Blood	Homs *et al.*	up	1	0
C2orf70	Autism-GSE83424_Blood	Homs *et al.*	up	0	1
CALHM2	Autism-GSE83424_Blood	Homs *et al.*	up	0	1
CALHM2	Autism-GSE83424_Blood	Homs *et al.*	up	0	1
CCDC169	Autism-GSE83424_Blood	Homs *et al.*	up	1	0
CCDC71L	Autism-GSE83424_Blood	Homs *et al.*	up	1	1
CKMT2-AS1	Autism-GSE83424_Blood	Homs *et al.*	up	1	0
CRACR2A	Autism-GSE83424_Blood	Homs *et al.*	up	1	0
CYP51A1	Autism-GSE83424_Blood	Homs *et al.*	up	0	1
CYP51A1-AS1	Autism-GSE83424_Blood	Homs *et al.*	up	0	1
DEPDC1	Autism-GSE83424_Blood	Homs *et al.*	up	0	1
DEPDC1-AS1	Autism-GSE83424_Blood	Homs *et al.*	up	0	1
FDFT1	Autism-GSE83424_Blood	Homs *et al.*	up	1	0
FLJ30679	Autism-GSE83424_Blood	Homs *et al.*	up	1	0
FRA10AC1	Autism-GSE83424_Blood	Homs *et al.*	up	1	0

Gene Symbol	Dataset	Author	Up/Down Methylated	#Cases	# Controls
GOPC	Autism-GSE83424_Blood	Homs *et al.*	up	1	0
HOXA4	Autism-GSE83424_Blood	Homs *et al.*	up	0	1
HOXA7	Autism-GSE83424_Blood	Homs *et al.*	up	1	0
HOXA7	Autism-GSE83424_Blood	Homs *et al.*	up	1	0
IFITM3	Autism-GSE83424_Blood	Homs *et al.*	up	1	0
ISOC2	Autism-GSE83424_Blood	Homs *et al.*	up	1	1
KATNB1	Autism-GSE83424_Blood	Homs *et al.*	up	1	0
LINC00941	Autism-GSE83424_Blood	Homs *et al.*	up	0	1
LMCD1-AS1	Autism-GSE83424_Blood	Homs *et al.*	up	1	0
LMCD1	Autism-GSE83424_Blood	Homs *et al.*	up	1	0
LOC285804	Autism-GSE83424_Blood	Homs *et al.*	up	0	1
LOC400710	Autism-GSE83424_Blood	Homs *et al.*	up	1	0
LRRC61	Autism-GSE83424_Blood	Homs *et al.*	up	0	1
MESTIT1	Autism-GSE83424_Blood	Homs *et al.*	up	1	0
MFSD4A	Autism-GSE83424_Blood	Homs *et al.*	up	1	0
MIR7159	Autism-GSE83424_Blood	Homs *et al.*	up	1	0
NEDD4L	Autism-GSE83424_Blood	Homs *et al.*	up	1	0
PNPO	Autism-GSE83424_Blood	Homs *et al.*	up	0	1
PNPO	Autism-GSE83424_Blood	Homs *et al.*	up	0	1
POLR3K	Autism-GSE83424_Blood	Homs *et al.*	up	1	0
POLR3K	Autism-GSE83424_Blood	Homs *et al.*	up	1	0
RNU5D-1	Autism-GSE83424_Blood	Homs *et al.*	up	1	0
S100A6	Autism-GSE83424_Blood	Homs *et al.*	up	0	1
S100A6	Autism-GSE83424_Blood	Homs *et al.*	up	0	1
TCF19	Autism-GSE83424_Blood	Homs *et al.*	up	1	0
TRIM31-AS1	Autism-GSE83424_Blood	Homs *et al.*	up	0	1
TRIM39-RPP21	Autism-GSE83424_Blood	Homs *et al.*	up	1	1
TRIM4	Autism-GSE83424_Blood	Homs *et al.*	up	1	0
TRIM61	Autism-GSE83424_Blood	Homs *et al.*	up	0	1
TSTD1	Autism-GSE83424_Blood	Homs *et al.*	up	1	0
TSTD1	Autism-GSE83424_Blood	Homs *et al.*	up	1	0
ZNF304	Autism-GSE83424_Blood	Homs *et al.*	up	0	1
ZNF382	Autism-GSE83424_Blood	Homs *et al.*	up	0	1
ZNF529	Autism-GSE83424_Blood	Homs *et al.*	up	0	1
ZNF552	Autism-GSE83424_Blood	Homs *et al.*	up	1	0
ZNF562	Autism-GSE83424_Blood	Homs *et al.*	up	1	0
B4GALNT4	Autism-GSE100905_Blood	Kimura *et al.*	down	0	1
CASZ1	Autism-GSE100905_Blood	Kimura *et al.*	down	0	1
CD163	Autism-GSE100905_Blood	Kimura *et al.*	down	0	1
COLEC11	Autism-GSE100905_Blood	Kimura *et al.*	down	1	1
DIP2C	Autism-GSE100905_Blood	Kimura *et al.*	down	1	1
DUSP22	Autism-GSE100905_Blood	Kimura *et al.*	down	1	1
ELOA2	Autism-GSE100905_Blood	Kimura *et al.*	down	1	1
FZD1	Autism-GSE100905_Blood	Kimura *et al.*	down	1	0
IGFALS	Autism-GSE100905_Blood	Kimura *et al.*	down	0	1
IGFALS	Autism-GSE100905_Blood	Kimura *et al.*	down	1	1

Gene Symbol	Dataset	Author	Up/Down Methylated	#Cases	# Controls
KATNAL2	Autism-GSE100905_Blood	Kimura *et al.*	down	1	1
LDHC	Autism-GSE100905_Blood	Kimura *et al.*	down	1	0
LINC01101	Autism-GSE100905_Blood	Kimura *et al.*	down	1	0
LINC01623	Autism-GSE100905_Blood	Kimura *et al.*	down	0	1
MEG8	Autism-GSE100905_Blood	Kimura *et al.*	down	0	1
RPH3AL	Autism-GSE100905_Blood	Kimura *et al.*	down	0	1
STPG4	Autism-GSE100905_Blood	Kimura *et al.*	down	1	1
ZDHHC14	Autism-GSE100905_Blood	Kimura *et al.*	down	0	1
ACTR3C	Autism-GSE100905_Blood	Kimura *et al.*	up	1	0
AGPAT1	Autism-GSE100905_Blood	Kimura *et al.*	up	0	1
ALKBH3	Autism-GSE100905_Blood	Kimura *et al.*	up	0	1
BOLL	Autism-GSE100905_Blood	Kimura *et al.*	up	1	0
C6orf48	Autism-GSE100905_Blood	Kimura *et al.*	up	1	0
CABYR	Autism-GSE100905_Blood	Kimura *et al.*	up	1	0
CALCA	Autism-GSE100905_Blood	Kimura *et al.*	up	0	1
CAMLG	Autism-GSE100905_Blood	Kimura *et al.*	up	0	1
CAT	Autism-GSE100905_Blood	Kimura *et al.*	up	0	1
CCDC127	Autism-GSE100905_Blood	Kimura *et al.*	up	1	0
CCDC169	Autism-GSE100905_Blood	Kimura *et al.*	up	1	1
COX18	Autism-GSE100905_Blood	Kimura *et al.*	up	0	1
CPEB1	Autism-GSE100905_Blood	Kimura *et al.*	up	0	1
CR1L	Autism-GSE100905_Blood	Kimura *et al.*	up	0	1
CYP46A1	Autism-GSE100905_Blood	Kimura *et al.*	up	1	0
DNAH12	Autism-GSE100905_Blood	Kimura *et al.*	up	0	1
DUSP19	Autism-GSE100905_Blood	Kimura *et al.*	up	0	1
EDNRB	Autism-GSE100905_Blood	Kimura *et al.*	up	1	0
EFCAB10	Autism-GSE100905_Blood	Kimura *et al.*	up	1	0
EHHADH	Autism-GSE100905_Blood	Kimura *et al.*	up	0	1
EIPR1	Autism-GSE100905_Blood	Kimura *et al.*	up	0	1
EXOC3L4	Autism-GSE100905_Blood	Kimura *et al.*	up	0	1
FAM193B	Autism-GSE100905_Blood	Kimura *et al.*	up	0	1
FAM218A	Autism-GSE100905_Blood	Kimura *et al.*	up	0	1
FBXL16	Autism-GSE100905_Blood	Kimura *et al.*	up	1	0
FDFT1	Autism-GSE100905_Blood	Kimura *et al.*	up	1	1
FKBP11	Autism-GSE100905_Blood	Kimura *et al.*	up	1	0
FOSB	Autism-GSE100905_Blood	Kimura *et al.*	up	1	0
GSDMD	Autism-GSE100905_Blood	Kimura *et al.*	up	0	1
HCG27	Autism-GSE100905_Blood	Kimura *et al.*	up	1	0
HGSNAT	Autism-GSE100905_Blood	Kimura *et al.*	up	1	0
ISOC2	Autism-GSE100905_Blood	Kimura *et al.*	up	1	1
KATNB1	Autism-GSE100905_Blood	Kimura *et al.*	up	1	0
KIF6	Autism-GSE100905_Blood	Kimura *et al.*	up	1	0
KPNA5	Autism-GSE100905_Blood	Kimura *et al.*	up	1	0
LDAH	Autism-GSE100905_Blood	Kimura *et al.*	up	1	0
LINC01685	Autism-GSE100905_Blood	Kimura *et al.*	up	1	0
LINC01725	Autism-GSE100905_Blood	Kimura *et al.*	up	1	0

Gene Symbol	Dataset	Author	Up/Down Methylated	#Cases	# Controls
LOC101927560	Autism-GSE100905_Blood	Kimura *et al.*	up	1	0
LOC105371430	Autism-GSE100905_Blood	Kimura *et al.*	up	1	0
LOC105378683	Autism-GSE100905_Blood	Kimura *et al.*	up	1	0
LOC284798	Autism-GSE100905_Blood	Kimura *et al.*	up	1	1
LRRC61	Autism-GSE100905_Blood	Kimura *et al.*	up	1	0
LRTM2	Autism-GSE100905_Blood	Kimura *et al.*	up	1	0
MAST1	Autism-GSE100905_Blood	Kimura *et al.*	up	1	0
MAST4	Autism-GSE100905_Blood	Kimura *et al.*	up	1	0
MEDAG	Autism-GSE100905_Blood	Kimura *et al.*	up	0	1
MEST	Autism-GSE100905_Blood	Kimura *et al.*	up	1	0
MIR596	Autism-GSE100905_Blood	Kimura *et al.*	up	0	1
MTERF1	Autism-GSE100905_Blood	Kimura *et al.*	up	0	1
NEFM	Autism-GSE100905_Blood	Kimura *et al.*	up	1	0
NKX1-1	Autism-GSE100905_Blood	Kimura *et al.*	up	1	1
NKX2-6	Autism-GSE100905_Blood	Kimura *et al.*	up	0	1
NMU	Autism-GSE100905_Blood	Kimura *et al.*	up	1	0
NONE	Autism-GSE100905_Blood	Kimura *et al.*	up	1	0
NPY	Autism-GSE100905_Blood	Kimura *et al.*	up	1	0
OR4D9	Autism-GSE100905_Blood	Kimura *et al.*	up	1	0
PACS2	Autism-GSE100905_Blood	Kimura *et al.*	up	0	1
PAXIP1-AS2	Autism-GSE100905_Blood	Kimura *et al.*	up	1	0
PDE4A	Autism-GSE100905_Blood	Kimura *et al.*	up	1	1
PHYHIP	Autism-GSE100905_Blood	Kimura *et al.*	up	0	1
PLSCR1	Autism-GSE100905_Blood	Kimura *et al.*	up	0	1
PON3	Autism-GSE100905_Blood	Kimura *et al.*	up	1	0
RAET1L	Autism-GSE100905_Blood	Kimura *et al.*	up	1	0
RGS7	Autism-GSE100905_Blood	Kimura *et al.*	up	1	0
RNF5P1	Autism-GSE100905_Blood	Kimura *et al.*	up	1	1
S100A6	Autism-GSE100905_Blood	Kimura *et al.*	up	1	0
S100A6	Autism-GSE100905_Blood	Kimura *et al.*	up	1	0
SDHA	Autism-GSE100905_Blood	Kimura *et al.*	up	1	0
LOC44813	Autism-GSE100905_Blood	Kimura *et al.*	up	1	1
SHANK2	Autism-GSE100905_Blood	Kimura *et al.*	up	0	1
SVIL-AS1	Autism-GSE100905_Blood	Kimura *et al.*	up	0	1
TEX14	Autism-GSE100905_Blood	Kimura *et al.*	up	1	0
TEX26-AS1	Autism-GSE100905_Blood	Kimura *et al.*	up	0	1
TMEM161B-AS1	Autism-GSE100905_Blood	Kimura *et al.*	up	0	1
TREX1	Autism-GSE100905_Blood	Kimura *et al.*	up	1	1
TRIM39-RPP21	Autism-GSE100905_Blood	Kimura *et al.*	up	1	0
TRIM4	Autism-GSE100905_Blood	Kimura *et al.*	up	1	0
TRIM6	Autism-GSE100905_Blood	Kimura *et al.*	up	0	1
TRIM6	Autism-GSE100905_Blood	Kimura *et al.*	up	0	1
TRIM61	Autism-GSE100905_Blood	Kimura *et al.*	up	0	1
TRMT12	Autism-GSE100905_Blood	Kimura *et al.*	up	0	1
TTLL10	Autism-GSE100905_Blood	Kimura *et al.*	up	0	1
VENTX	Autism-GSE100905_Blood	Kimura *et al.*	up	1	1

Gene Symbol	Dataset	Author	Up/Down Methylated	#Cases	# Controls
ZNF300P1	Autism-GSE100905_Blood	Kimura *et al.*	up	1	0
ZNF33B	Autism-GSE100905_Blood	Kimura *et al.*	up	0	1
ZNF511	Autism-GSE100905_Blood	Kimura *et al.*	up	1	0
ZNF561	Autism-GSE100905_Blood	Kimura *et al.*	up	1	0
ZNF562	Autism-GSE100905_Blood	Kimura *et al.*	up	1	1
ZNF681	Autism-GSE100905_Blood	Kimura *et al.*	up	1	1
ZSCAN31	Autism-GSE100905_Blood	Kimura *et al.*	up	0	1
ACY3	Autism-GSE113967_blood	Siu *et al.*	down	1	1
ADAMTS4	Autism-GSE113967_blood	Siu *et al.*	down	1	0
ADAMTS4	Autism-GSE113967_blood	Siu *et al.*	down	1	0
ADCY10P1	Autism-GSE113967_blood	Siu *et al.*	down	0	1
BOLL	Autism-GSE113967_blood	Siu *et al.*	down	0	1
CMYA5	Autism-GSE113967_blood	Siu *et al.*	down	1	1
CNTNAP1	Autism-GSE113967_blood	Siu *et al.*	down	0	1
COL18A1	Autism-GSE113967_blood	Siu *et al.*	down	1	0
COL4A1	Autism-GSE113967_blood	Siu *et al.*	down	0	1
DIP2C	Autism-GSE113967_blood	Siu *et al.*	down	0	1
DNAH17	Autism-GSE113967_blood	Siu *et al.*	down	1	0
DUSP22	Autism-GSE113967_blood	Siu *et al.*	down	1	1
DVL3	Autism-GSE113967_blood	Siu *et al.*	down	0	1
ELOA2	Autism-GSE113967_blood	Siu *et al.*	down	1	0
EXOC2	Autism-GSE113967_blood	Siu *et al.*	down	1	0
FAM83A	Autism-GSE113967_blood	Siu *et al.*	down	0	1
FOXF2	Autism-GSE113967_blood	Siu *et al.*	down	1	0
GCNT2	Autism-GSE113967_blood	Siu *et al.*	down	1	0
GFI1	Autism-GSE113967_blood	Siu *et al.*	down	1	1
GMDS	Autism-GSE113967_blood	Siu *et al.*	down	1	0
GNG12-AS1	Autism-GSE113967_blood	Siu *et al.*	down	1	0
GPHA2	Autism-GSE113967_blood	Siu *et al.*	down	0	1
GPR37L1	Autism-GSE113967_blood	Siu *et al.*	down	1	0
HAGH	Autism-GSE113967_blood	Siu *et al.*	down	0	1
HS3ST3B1	Autism-GSE113967_blood	Siu *et al.*	down	1	0
HUS1B	Autism-GSE113967_blood	Siu *et al.*	down	1	0
IGFALS	Autism-GSE113967_blood	Siu *et al.*	down	0	1
KATNAL2	Autism-GSE113967_blood	Siu *et al.*	down	1	0
KCNAB3	Autism-GSE113967_blood	Siu *et al.*	down	0	1
KCNAB3	Autism-GSE113967_blood	Siu *et al.*	down	0	1
KCNQ1	Autism-GSE113967_blood	Siu *et al.*	down	0	1
KLHDC7A	Autism-GSE113967_blood	Siu *et al.*	down	0	1
LDHC	Autism-GSE113967_blood	Siu *et al.*	down	1	0
LINC00982	Autism-GSE113967_blood	Siu *et al.*	down	1	0
LINC01102	Autism-GSE113967_blood	Siu *et al.*	down	0	1
LINC02470	Autism-GSE113967_blood	Siu *et al.*	down	0	1
LOC100507547	Autism-GSE113967_blood	Siu *et al.*	down	0	1
LOC102724511	Autism-GSE113967_blood	Siu *et al.*	down	1	1
LOC105378127	Autism-GSE113967_blood	Siu *et al.*	down	1	0

Gene Symbol	Dataset	Author	Up/Down Methylated	#Cases	# Controls
LRAT	Autism-GSE113967_blood	Siu *et al.*	down	1	0
LTA	Autism-GSE113967_blood	Siu *et al.*	down	1	0
MAD1L1	Autism-GSE113967_blood	Siu *et al.*	down	1	0
MBP	Autism-GSE113967_blood	Siu *et al.*	down	1	0
MCF2L	Autism-GSE113967_blood	Siu *et al.*	down	0	1
MGC12916	Autism-GSE113967_blood	Siu *et al.*	down	1	0
MINCR	Autism-GSE113967_blood	Siu *et al.*	down	0	1
MIR1268A	Autism-GSE113967_blood	Siu *et al.*	down	1	0
MIR219A2	Autism-GSE113967_blood	Siu *et al.*	down	1	0
MIR219A2	Autism-GSE113967_blood	Siu *et al.*	down	1	0
MIR662	Autism-GSE113967_blood	Siu *et al.*	down	1	0
MSH4	Autism-GSE113967_blood	Siu *et al.*	down	0	1
NCR2	Autism-GSE113967_blood	Siu *et al.*	down	0	1
NFYA	Autism-GSE113967_blood	Siu *et al.*	down	1	1
NOC2L	Autism-GSE113967_blood	Siu *et al.*	down	0	1
NUP210L	Autism-GSE113967_blood	Siu *et al.*	down	1	1
PAPD7	Autism-GSE113967_blood	Siu *et al.*	down	1	0
PNLDC1	Autism-GSE113967_blood	Siu *et al.*	down	1	0
PPIEL	Autism-GSE113967_blood	Siu *et al.*	down	1	0
PRRT1	Autism-GSE113967_blood	Siu *et al.*	down	1	1
PRSS42	Autism-GSE113967_blood	Siu *et al.*	down	1	1
RAD51C	Autism-GSE113967_blood	Siu *et al.*	down	1	0
RPH3AL	Autism-GSE113967_blood	Siu *et al.*	down	0	1
SAMD11	Autism-GSE113967_blood	Siu *et al.*	down	0	1
SNU13	Autism-GSE113967_blood	Siu *et al.*	down	1	0
STPG4	Autism-GSE113967_blood	Siu *et al.*	down	1	0
TCIRG1	Autism-GSE113967_blood	Siu *et al.*	down	0	1
TEX14	Autism-GSE113967_blood	Siu *et al.*	down	1	0
TMEM163	Autism-GSE113967_blood	Siu *et al.*	down	0	1
TRAPPC12	Autism-GSE113967_blood	Siu *et al.*	down	0	1
TRIM31-AS1	Autism-GSE113967_blood	Siu *et al.*	down	0	1
UBE2U	Autism-GSE113967_blood	Siu *et al.*	down	0	1
UPP1	Autism-GSE113967_blood	Siu *et al.*	down	0	1
VTRNA2-1	Autism-GSE113967_blood	Siu *et al.*	down	1	1
WDR97	Autism-GSE113967_blood	Siu *et al.*	down	1	1
WDR97	Autism-GSE113967_blood	Siu *et al.*	down	0	1
WFIKKN2	Autism-GSE113967_blood	Siu *et al.*	down	0	1
ZDHHC14	Autism-GSE113967_blood	Siu *et al.*	down	0	1
ACP2	Autism-GSE113967_blood	Siu *et al.*	up	1	0
ACP2	Autism-GSE113967_blood	Siu *et al.*	up	1	0
ACTR3C	Autism-GSE113967_blood	Siu *et al.*	up	1	1
AGBL3	Autism-GSE113967_blood	Siu *et al.*	up	0	1
AGPAT1	Autism-GSE113967_blood	Siu *et al.*	up	1	1
AGPAT1	Autism-GSE113967_blood	Siu *et al.*	up	1	1
AIF1	Autism-GSE113967_blood	Siu *et al.*	up	1	0
AKAP12	Autism-GSE113967_blood	Siu *et al.*	up	1	1

Gene Symbol	Dataset	Author	Up/Down Methylated	#Cases	# Controls
ALKBH3	Autism-GSE113967_blood	Siu *et al.*	up	1	1
ARHGAP22	Autism-GSE113967_blood	Siu *et al.*	up	1	0
ASTE1	Autism-GSE113967_blood	Siu *et al.*	up	1	0
BCAN	Autism-GSE113967_blood	Siu *et al.*	up	1	0
BMP4	Autism-GSE113967_blood	Siu *et al.*	up	1	0
C11orf87	Autism-GSE113967_blood	Siu *et al.*	up	1	0
C2CD4B	Autism-GSE113967_blood	Siu *et al.*	up	0	1
C2CD4B	Autism-GSE113967_blood	Siu *et al.*	up	0	1
C2orf70	Autism-GSE113967_blood	Siu *et al.*	up	1	1
C6orf48	Autism-GSE113967_blood	Siu *et al.*	up	1	1
C7orf50	Autism-GSE113967_blood	Siu *et al.*	up	1	0
CACNA2D4	Autism-GSE113967_blood	Siu *et al.*	up	0	1
CALCB	Autism-GSE113967_blood	Siu *et al.*	up	1	0
CALHM2	Autism-GSE113967_blood	Siu *et al.*	up	1	1
CCDC169	Autism-GSE113967_blood	Siu *et al.*	up	1	1
CCDC181	Autism-GSE113967_blood	Siu *et al.*	up	0	1
CCDC71L	Autism-GSE113967_blood	Siu *et al.*	up	1	1
CCK	Autism-GSE113967_blood	Siu *et al.*	up	1	0
CCNG1	Autism-GSE113967_blood	Siu *et al.*	up	1	0
CCT5	Autism-GSE113967_blood	Siu *et al.*	up	0	1
CD8B2	Autism-GSE113967_blood	Siu *et al.*	up	1	1
CDK1	Autism-GSE113967_blood	Siu *et al.*	up	1	0
CENPF	Autism-GSE113967_blood	Siu *et al.*	up	1	1
CFAP73	Autism-GSE113967_blood	Siu *et al.*	up	0	1
CHMP4C	Autism-GSE113967_blood	Siu *et al.*	up	0	1
CMTM2	Autism-GSE113967_blood	Siu *et al.*	up	1	0
CMTM2	Autism-GSE113967_blood	Siu *et al.*	up	1	1
CNR2	Autism-GSE113967_blood	Siu *et al.*	up	1	0
COMT	Autism-GSE113967_blood	Siu *et al.*	up	1	0
COPG1	Autism-GSE113967_blood	Siu *et al.*	up	1	0
CPEB1	Autism-GSE113967_blood	Siu *et al.*	up	1	0
CRACR2A	Autism-GSE113967_blood	Siu *et al.*	up	1	1
CREB3L3	Autism-GSE113967_blood	Siu *et al.*	up	1	1
CRYZ	Autism-GSE113967_blood	Siu *et al.*	up	1	1
CRYZ	Autism-GSE113967_blood	Siu *et al.*	up	1	1
CTHRC1	Autism-GSE113967_blood	Siu *et al.*	up	1	0
CTSF	Autism-GSE113967_blood	Siu *et al.*	up	1	0
CWH43	Autism-GSE113967_blood	Siu *et al.*	up	0	1
CYP51A1-AS1	Autism-GSE113967_blood	Siu *et al.*	up	0	1
DDX56	Autism-GSE113967_blood	Siu *et al.*	up	1	0
DDX60	Autism-GSE113967_blood	Siu *et al.*	up	1	0
DENND3	Autism-GSE113967_blood	Siu *et al.*	up	1	1
DGKG	Autism-GSE113967_blood	Siu *et al.*	up	0	1
DHFR2	Autism-GSE113967_blood	Siu *et al.*	up	0	1
DIP2B	Autism-GSE113967_blood	Siu *et al.*	up	1	0
DIRAS3	Autism-GSE113967_blood	Siu *et al.*	up	0	1

Gene Symbol	Dataset	Author	Up/Down Methylated	#Cases	# Controls
DMRT3	Autism-GSE113967_blood	Siu *et al.*	up	1	0
DNMT3A	Autism-GSE113967_blood	Siu *et al.*	up	0	1
DUOXA1	Autism-GSE113967_blood	Siu *et al.*	up	1	0
DUOXA1	Autism-GSE113967_blood	Siu *et al.*	up	1	0
DUOXA2	Autism-GSE113967_blood	Siu *et al.*	up	1	0
ECHDC1	Autism-GSE113967_blood	Siu *et al.*	up	1	0
EFCAB10	Autism-GSE113967_blood	Siu *et al.*	up	1	0
EIPR1	Autism-GSE113967_blood	Siu *et al.*	up	1	0
ELF1	Autism-GSE113967_blood	Siu *et al.*	up	0	1
ERCC8	Autism-GSE113967_blood	Siu *et al.*	up	1	0
ERCC8	Autism-GSE113967_blood	Siu *et al.*	up	1	0
ERGIC1	Autism-GSE113967_blood	Siu *et al.*	up	0	1
ETFB	Autism-GSE113967_blood	Siu *et al.*	up	1	0
FAM193B	Autism-GSE113967_blood	Siu *et al.*	up	0	1
FAR2	Autism-GSE113967_blood	Siu *et al.*	up	1	0
FDFT1	Autism-GSE113967_blood	Siu *et al.*	up	1	1
FEZF1-AS1	Autism-GSE113967_blood	Siu *et al.*	up	0	1
FKBP11	Autism-GSE113967_blood	Siu *et al.*	up	0	1
FKBP2	Autism-GSE113967_blood	Siu *et al.*	up	1	0
GANC	Autism-GSE113967_blood	Siu *et al.*	up	1	0
GAS2L2	Autism-GSE113967_blood	Siu *et al.*	up	0	1
GJB6	Autism-GSE113967_blood	Siu *et al.*	up	1	0
GPR180	Autism-GSE113967_blood	Siu *et al.*	up	1	1
GTF2IRD1	Autism-GSE113967_blood	Siu *et al.*	up	1	0
GTPBP3	Autism-GSE113967_blood	Siu *et al.*	up	0	1
HCG27	Autism-GSE113967_blood	Siu *et al.*	up	0	1
HDAC11-AS1	Autism-GSE113967_blood	Siu *et al.*	up	1	0
HELZ2	Autism-GSE113967_blood	Siu *et al.*	up	1	0
HIST1H2BB	Autism-GSE113967_blood	Siu *et al.*	up	0	1
HIST1H2BO	Autism-GSE113967_blood	Siu *et al.*	up	1	1
HIST1H3C	Autism-GSE113967_blood	Siu *et al.*	up	0	1
HLA-DPB2	Autism-GSE113967_blood	Siu *et al.*	up	1	1
HMOX1	Autism-GSE113967_blood	Siu *et al.*	up	0	1
HPDL	Autism-GSE113967_blood	Siu *et al.*	up	1	0
HPS3	Autism-GSE113967_blood	Siu *et al.*	up	0	1
HSD17B14	Autism-GSE113967_blood	Siu *et al.*	up	1	1
IRAK4	Autism-GSE113967_blood	Siu *et al.*	up	0	1
ISOC2	Autism-GSE113967_blood	Siu *et al.*	up	1	1
KATNB1	Autism-GSE113967_blood	Siu *et al.*	up	1	0
KBTBD11	Autism-GSE113967_blood	Siu *et al.*	up	0	1
KCNAB2	Autism-GSE113967_blood	Siu *et al.*	up	1	0
KCNJ10	Autism-GSE113967_blood	Siu *et al.*	up	0	1
KIF6	Autism-GSE113967_blood	Siu *et al.*	up	0	1
KLHL17	Autism-GSE113967_blood	Siu *et al.*	up	1	0
KRT222	Autism-GSE113967_blood	Siu *et al.*	up	0	1
LCLAT1	Autism-GSE113967_blood	Siu *et al.*	up	1	0

Gene Symbol	Dataset	Author	Up/Down Methylated	#Cases	# Controls
LINC00467	Autism-GSE113967_blood	Siu *et al.*	up	1	0
LINC00506	Autism-GSE113967_blood	Siu *et al.*	up	1	0
LINC00941	Autism-GSE113967_blood	Siu *et al.*	up	1	0
LINC01149	Autism-GSE113967_blood	Siu *et al.*	up	1	1
LINC01166	Autism-GSE113967_blood	Siu *et al.*	up	1	1
LINC01167	Autism-GSE113967_blood	Siu *et al.*	up	1	1
LINC01230	Autism-GSE113967_blood	Siu *et al.*	up	1	0
LINC01341	Autism-GSE113967_blood	Siu *et al.*	up	1	0
LINC01535	Autism-GSE113967_blood	Siu *et al.*	up	0	1
LINC01556	Autism-GSE113967_blood	Siu *et al.*	up	1	0
LINC01556	Autism-GSE113967_blood	Siu *et al.*	up	1	0
LINC01565	Autism-GSE113967_blood	Siu *et al.*	up	0	1
LINC01637	Autism-GSE113967_blood	Siu *et al.*	up	0	1
LINC01725	Autism-GSE113967_blood	Siu *et al.*	up	1	1
LINC01725	Autism-GSE113967_blood	Siu *et al.*	up	1	1
LINC01798	Autism-GSE113967_blood	Siu *et al.*	up	1	0
LINGO3	Autism-GSE113967_blood	Siu *et al.*	up	1	0
LNPK	Autism-GSE113967_blood	Siu *et al.*	up	1	1
LOC100126784	Autism-GSE113967_blood	Siu *et al.*	up	1	0
LOC100130417	Autism-GSE113967_blood	Siu *et al.*	up	1	0
LOC100133286	Autism-GSE113967_blood	Siu *et al.*	up	1	1
LOC101930114	Autism-GSE113967_blood	Siu *et al.*	up	1	0
LOC101930114	Autism-GSE113967_blood	Siu *et al.*	up	0	1
LOC105371430	Autism-GSE113967_blood	Siu *et al.*	up	1	1
LOC151174	Autism-GSE113967_blood	Siu *et al.*	up	1	1
LOC254028	Autism-GSE113967_blood	Siu *et al.*	up	1	0
LOC284798	Autism-GSE113967_blood	Siu *et al.*	up	1	0
LOC285804	Autism-GSE113967_blood	Siu *et al.*	up	1	0
LOC400710	Autism-GSE113967_blood	Siu *et al.*	up	1	1
LOC642366	Autism-GSE113967_blood	Siu *et al.*	up	1	0
LOC652276	Autism-GSE113967_blood	Siu *et al.*	up	1	1
LOC652276	Autism-GSE113967_blood	Siu *et al.*	up	1	1
LOC728392	Autism-GSE113967_blood	Siu *et al.*	up	1	0
LOR	Autism-GSE113967_blood	Siu *et al.*	up	1	0
LRRC14B	Autism-GSE113967_blood	Siu *et al.*	up	0	1
LRRC4C	Autism-GSE113967_blood	Siu *et al.*	up	0	1
LRRC61	Autism-GSE113967_blood	Siu *et al.*	up	1	1
LRTM2	Autism-GSE113967_blood	Siu *et al.*	up	0	1
LYPD5	Autism-GSE113967_blood	Siu *et al.*	up	1	0
MARVELD2	Autism-GSE113967_blood	Siu *et al.*	up	1	0
MAST1	Autism-GSE113967_blood	Siu *et al.*	up	0	1
MCCC1	Autism-GSE113967_blood	Siu *et al.*	up	1	0
MIR3649	Autism-GSE113967_blood	Siu *et al.*	up	0	1
MIR5188	Autism-GSE113967_blood	Siu *et al.*	up	1	0
MIR548H4	Autism-GSE113967_blood	Siu *et al.*	up	0	1
MIR596	Autism-GSE113967_blood	Siu *et al.*	up	1	1

Gene Symbol	Dataset	Author	Up/Down Methylated	#Cases	# Controls
MPDZ	Autism-GSE113967_blood	Siu *et al.*	up	1	0
MPHOSPH6	Autism-GSE113967_blood	Siu *et al.*	up	1	0
MPIG6B	Autism-GSE113967_blood	Siu *et al.*	up	1	0
MRPL35	Autism-GSE113967_blood	Siu *et al.*	up	0	1
MXRA8	Autism-GSE113967_blood	Siu *et al.*	up	0	1
NDUFAF2	Autism-GSE113967_blood	Siu *et al.*	up	1	0
NEAT1	Autism-GSE113967_blood	Siu *et al.*	up	0	1
NEK11	Autism-GSE113967_blood	Siu *et al.*	up	1	0
NKX1-1	Autism-GSE113967_blood	Siu *et al.*	up	0	1
NME3	Autism-GSE113967_blood	Siu *et al.*	up	1	0
NME3	Autism-GSE113967_blood	Siu *et al.*	up	1	0
NOC2L	Autism-GSE113967_blood	Siu *et al.*	up	1	0
NOX5	Autism-GSE113967_blood	Siu *et al.*	up	0	1
NPY	Autism-GSE113967_blood	Siu *et al.*	up	1	0
NR1H3	Autism-GSE113967_blood	Siu *et al.*	up	1	0
NUDT12	Autism-GSE113967_blood	Siu *et al.*	up	1	0
OR4D9	Autism-GSE113967_blood	Siu *et al.*	up	1	0
OXGR1	Autism-GSE113967_blood	Siu *et al.*	up	1	1
PACS2	Autism-GSE113967_blood	Siu *et al.*	up	1	1
PCTP	Autism-GSE113967_blood	Siu *et al.*	up	0	1
PDE4A	Autism-GSE113967_blood	Siu *et al.*	up	1	0
PDZD7	Autism-GSE113967_blood	Siu *et al.*	up	0	1
PHYHIP	Autism-GSE113967_blood	Siu *et al.*	up	1	0
PLD6	Autism-GSE113967_blood	Siu *et al.*	up	1	0
PLEKHA4	Autism-GSE113967_blood	Siu *et al.*	up	1	1
PLEKHN1	Autism-GSE113967_blood	Siu *et al.*	up	1	0
PLEKHN1	Autism-GSE113967_blood	Siu *et al.*	up	1	0
PNPO	Autism-GSE113967_blood	Siu *et al.*	up	1	1
PNPO	Autism-GSE113967_blood	Siu *et al.*	up	1	1
PON3	Autism-GSE113967_blood	Siu *et al.*	up	1	1
PRICKLE1	Autism-GSE113967_blood	Siu *et al.*	up	0	1
PRR15	Autism-GSE113967_blood	Siu *et al.*	up	1	0
PTPRN2	Autism-GSE113967_blood	Siu *et al.*	up	0	1
PUS7L	Autism-GSE113967_blood	Siu *et al.*	up	0	1
PXMP4	Autism-GSE113967_blood	Siu *et al.*	up	0	1
RAB26	Autism-GSE113967_blood	Siu *et al.*	up	1	0
RAB3GAP2	Autism-GSE113967_blood	Siu *et al.*	up	1	0
RASSF6	Autism-GSE113967_blood	Siu *et al.*	up	0	1
RBM43	Autism-GSE113967_blood	Siu *et al.*	up	1	0
REC8	Autism-GSE113967_blood	Siu *et al.*	up	1	0
RHPN1	Autism-GSE113967_blood	Siu *et al.*	up	1	0
RIPK4	Autism-GSE113967_blood	Siu *et al.*	up	0	1
RNF39	Autism-GSE113967_blood	Siu *et al.*	up	1	1
RNF39	Autism-GSE113967_blood	Siu *et al.*	up	1	1
RNF5	Autism-GSE113967_blood	Siu *et al.*	up	0	1
RNF5P1	Autism-GSE113967_blood	Siu *et al.*	up	1	1

Gene Symbol	Dataset	Author	Up/Down Methylated	#Cases	# Controls
RPL13	Autism-GSE113967_blood	Siu *et al.*	up	1	0
RPL22L1	Autism-GSE113967_blood	Siu *et al.*	up	1	0
RWDD2B	Autism-GSE113967_blood	Siu *et al.*	up	1	0
SAMD11	Autism-GSE113967_blood	Siu *et al.*	up	1	0
SCAND2P	Autism-GSE113967_blood	Siu *et al.*	up	0	1
SCART1	Autism-GSE113967_blood	Siu *et al.*	up	1	0
SELENOP	Autism-GSE113967_blood	Siu *et al.*	up	1	0
LOC44813	Autism-GSE113967_blood	Siu *et al.*	up	1	1
SHANK2	Autism-GSE113967_blood	Siu *et al.*	up	1	1
SLC12A9	Autism-GSE113967_blood	Siu *et al.*	up	0	1
SLC25A38	Autism-GSE113967_blood	Siu *et al.*	up	1	0
SLC26A4-AS1	Autism-GSE113967_blood	Siu *et al.*	up	1	0
SLC27A6	Autism-GSE113967_blood	Siu *et al.*	up	1	0
SLC45A4	Autism-GSE113967_blood	Siu *et al.*	up	1	1
SLFN12L	Autism-GSE113967_blood	Siu *et al.*	up	1	0
SLMO2-ATP5E	Autism-GSE113967_blood	Siu *et al.*	up	1	0
SOGA3	Autism-GSE113967_blood	Siu *et al.*	up	1	0
SORBS2	Autism-GSE113967_blood	Siu *et al.*	up	1	0
SORD	Autism-GSE113967_blood	Siu *et al.*	up	1	1
SPAG1	Autism-GSE113967_blood	Siu *et al.*	up	1	1
SPAG17	Autism-GSE113967_blood	Siu *et al.*	up	0	1
SPAG8	Autism-GSE113967_blood	Siu *et al.*	up	0	1
SPAG8	Autism-GSE113967_blood	Siu *et al.*	up	0	1
SPATS1	Autism-GSE113967_blood	Siu *et al.*	up	0	1
SPON2	Autism-GSE113967_blood	Siu *et al.*	up	1	0
STAC	Autism-GSE113967_blood	Siu *et al.*	up	1	0
TACR3	Autism-GSE113967_blood	Siu *et al.*	up	1	1
TCIRG1	Autism-GSE113967_blood	Siu *et al.*	up	0	1
THSD7A	Autism-GSE113967_blood	Siu *et al.*	up	1	0
TMEM161B-AS1	Autism-GSE113967_blood	Siu *et al.*	up	1	0
TNFAIP8	Autism-GSE113967_blood	Siu *et al.*	up	0	1
TNFRSF9	Autism-GSE113967_blood	Siu *et al.*	up	0	1
TNIP2	Autism-GSE113967_blood	Siu *et al.*	up	1	0
TREX1	Autism-GSE113967_blood	Siu *et al.*	up	0	1
TRIM39-RPP21	Autism-GSE113967_blood	Siu *et al.*	up	1	0
TRIM4	Autism-GSE113967_blood	Siu *et al.*	up	0	1
TRIM6	Autism-GSE113967_blood	Siu *et al.*	up	1	1
TRIM6	Autism-GSE113967_blood	Siu *et al.*	up	1	1
TRIM61	Autism-GSE113967_blood	Siu *et al.*	up	1	0
TRMT12	Autism-GSE113967_blood	Siu *et al.*	up	1	1
TTLL13P	Autism-GSE113967_blood	Siu *et al.*	up	1	1
TUBGCP5	Autism-GSE113967_blood	Siu *et al.*	up	0	1
TXNRD2	Autism-GSE113967_blood	Siu *et al.*	up	1	1
TYW3	Autism-GSE113967_blood	Siu *et al.*	up	1	1
UBE2T	Autism-GSE113967_blood	Siu *et al.*	up	1	0
VMO1	Autism-GSE113967_blood	Siu *et al.*	up	0	1

Gene Symbol	Dataset	Author	Up/Down Methylated	#Cases	# Controls
VSTM5	Autism-GSE113967_blood	Siu *et al.*	up	1	1
VWA7	Autism-GSE113967_blood	Siu *et al.*	up	1	1
WNK4	Autism-GSE113967_blood	Siu *et al.*	up	1	0
WNT6	Autism-GSE113967_blood	Siu *et al.*	up	1	0
YOD1	Autism-GSE113967_blood	Siu *et al.*	up	1	0
ZBED3	Autism-GSE113967_blood	Siu *et al.*	up	0	1
ZBED9	Autism-GSE113967_blood	Siu *et al.*	up	1	0
ZFHX3	Autism-GSE113967_blood	Siu *et al.*	up	1	1
ZFP42	Autism-GSE113967_blood	Siu *et al.*	up	1	0
ZMAT2	Autism-GSE113967_blood	Siu *et al.*	up	0	1
ZMAT2	Autism-GSE113967_blood	Siu *et al.*	up	0	1
ZNF177	Autism-GSE113967_blood	Siu *et al.*	up	1	0
ZNF177	Autism-GSE113967_blood	Siu *et al.*	up	1	0
ZNF184	Autism-GSE113967_blood	Siu *et al.*	up	1	0
ZNF273	Autism-GSE113967_blood	Siu *et al.*	up	0	1
ZNF300P1	Autism-GSE113967_blood	Siu *et al.*	up	1	0
ZNF33B	Autism-GSE113967_blood	Siu *et al.*	up	1	0
ZNF382	Autism-GSE113967_blood	Siu *et al.*	up	1	0
ZNF529	Autism-GSE113967_blood	Siu *et al.*	up	1	0
ZNF559-ZNF177	Autism-GSE113967_blood	Siu *et al.*	up	1	0
ZNF561	Autism-GSE113967_blood	Siu *et al.*	up	1	1
ZNF561-AS1	Autism-GSE113967_blood	Siu *et al.*	up	1	1
ZNF561	Autism-GSE113967_blood	Siu *et al.*	up	1	1
ZNF563	Autism-GSE113967_blood	Siu *et al.*	up	1	0
ZNF57	Autism-GSE113967_blood	Siu *et al.*	up	1	1
ZNF681	Autism-GSE113967_blood	Siu *et al.*	up	0	1
ZNF703	Autism-GSE113967_blood	Siu *et al.*	up	0	1
ZNF772	Autism-GSE113967_blood	Siu *et al.*	up	1	0
ZNF814	Autism-GSE113967_blood	Siu *et al.*	up	0	1
ZNF833P	Autism-GSE113967_blood	Siu *et al.*	up	1	0
ZSWIM2	Autism-GSE113967_blood	Siu *et al.*	up	0	1
ATP11A	Autism-GSE131706_brain	Corley *et al.*	down	1	1
CCDC185	Autism-GSE131706_brain	Corley *et al.*	down	0	1
DUSP22	Autism-GSE131706_brain	Corley *et al.*	down	1	1
INPP5A	Autism-GSE131706_brain	Corley *et al.*	down	1	1
PAX8	Autism-GSE131706_brain	Corley *et al.*	down	1	0
PAX8-AS1	Autism-GSE131706_brain	Corley *et al.*	down	1	0
RPL10L	Autism-GSE131706_brain	Corley *et al.*	down	1	0
SFMBT1	Autism-GSE131706_brain	Corley *et al.*	down	0	1
TGFBI	Autism-GSE131706_brain	Corley *et al.*	down	1	0
VTRNA2-1	Autism-GSE131706_brain	Corley *et al.*	down	1	1
AGPAT1	Autism-GSE131706_brain	Corley *et al.*	up	1	0
AGPAT1	Autism-GSE131706_brain	Corley *et al.*	up	1	0
ALKBH3	Autism-GSE131706_brain	Corley *et al.*	up	1	0
ARHGAP22	Autism-GSE131706_brain	Corley *et al.*	up	1	1
BDNF-AS	Autism-GSE131706_brain	Corley *et al.*	up	0	1

Gene Symbol	Dataset	Author	Up/Down Methylated	#Cases	# Controls
C5orf63	Autism-GSE131706_brain	Corley *et al.*	up	0	1
CCDC169	Autism-GSE131706_brain	Corley *et al.*	up	1	0
CNPY3-GNMT	Autism-GSE131706_brain	Corley *et al.*	up	1	1
CRYBG1	Autism-GSE131706_brain	Corley *et al.*	up	1	1
ELOVL1	Autism-GSE131706_brain	Corley *et al.*	up	1	1
FAM218A	Autism-GSE131706_brain	Corley *et al.*	up	0	1
FAR2	Autism-GSE131706_brain	Corley *et al.*	up	1	0
FDFT1	Autism-GSE131706_brain	Corley *et al.*	up	1	0
FRG1	Autism-GSE131706_brain	Corley *et al.*	up	0	1
GRIK2	Autism-GSE131706_brain	Corley *et al.*	up	1	0
GSC	Autism-GSE131706_brain	Corley *et al.*	up	1	1
GTPBP3	Autism-GSE131706_brain	Corley *et al.*	up	1	0
HJURP	Autism-GSE131706_brain	Corley *et al.*	up	1	0
HOXA4	Autism-GSE131706_brain	Corley *et al.*	up	0	1
HSPA1B	Autism-GSE131706_brain	Corley *et al.*	up	0	1
ISOC2	Autism-GSE131706_brain	Corley *et al.*	up	1	1
KBTBD11	Autism-GSE131706_brain	Corley *et al.*	up	1	0
LIN7C	Autism-GSE131706_brain	Corley *et al.*	up	0	1
LINC00921	Autism-GSE131706_brain	Corley *et al.*	up	1	0
LINC01126	Autism-GSE131706_brain	Corley *et al.*	up	1	1
LINGO3	Autism-GSE131706_brain	Corley *et al.*	up	1	0
LOC400710	Autism-GSE131706_brain	Corley *et al.*	up	1	1
LY6G5C	Autism-GSE131706_brain	Corley *et al.*	up	1	1
LY6G5C	Autism-GSE131706_brain	Corley *et al.*	up	1	1
MSX1	Autism-GSE131706_brain	Corley *et al.*	up	1	1
OXGR1	Autism-GSE131706_brain	Corley *et al.*	up	0	1
PLD6	Autism-GSE131706_brain	Corley *et al.*	up	1	0
PRDM13	Autism-GSE131706_brain	Corley *et al.*	up	0	1
PRR15	Autism-GSE131706_brain	Corley *et al.*	up	0	1
PXMP4	Autism-GSE131706_brain	Corley *et al.*	up	0	1
RIN1	Autism-GSE131706_brain	Corley *et al.*	up	1	0
RNF39	Autism-GSE131706_brain	Corley *et al.*	up	1	1
RNF39	Autism-GSE131706_brain	Corley *et al.*	up	1	1
RNF5P1	Autism-GSE131706_brain	Corley *et al.*	up	1	0
RSPO4	Autism-GSE131706_brain	Corley *et al.*	up	1	0
SHANK2	Autism-GSE131706_brain	Corley *et al.*	up	1	0
SLC29A1	Autism-GSE131706_brain	Corley *et al.*	up	0	1
SLC29A1	Autism-GSE131706_brain	Corley *et al.*	up	0	1
SMARCD3	Autism-GSE131706_brain	Corley *et al.*	up	1	1
SOCS1	Autism-GSE131706_brain	Corley *et al.*	up	1	1
SOCS1	Autism-GSE131706_brain	Corley *et al.*	up	1	1
SORD	Autism-GSE131706_brain	Corley *et al.*	up	1	0
SOX2-OT	Autism-GSE131706_brain	Corley *et al.*	up	0	1
SPG20	Autism-GSE131706_brain	Corley *et al.*	up	0	1
TMEM33	Autism-GSE131706_brain	Corley *et al.*	up	1	0
TREX1	Autism-GSE131706_brain	Corley *et al.*	up	1	1

Gene Symbol	Dataset	Author	Up/Down Methylated	#Cases	# Controls
TRIM6	Autism-GSE131706_brain	Corley *et al.*	up	1	0
TRIM6	Autism-GSE131706_brain	Corley *et al.*	up	1	0
TRIM61	Autism-GSE131706_brain	Corley *et al.*	up	0	1
TUBGCP5	Autism-GSE131706_brain	Corley *et al.*	up	1	0
UNC45A	Autism-GSE131706_brain	Corley *et al.*	up	1	0
UNC45A	Autism-GSE131706_brain	Corley *et al.*	up	1	0
WNK4	Autism-GSE131706_brain	Corley *et al.*	up	1	0
WNT1	Autism-GSE131706_brain	Corley *et al.*	up	1	1
ZNF300	Autism-GSE131706_brain	Corley *et al.*	up	0	1
ZNF300P1	Autism-GSE131706_brain	Corley *et al.*	up	0	1
ZNF418	Autism-GSE131706_brain	Corley *et al.*	up	0	1
ZNF562	Autism-GSE131706_brain	Corley *et al.*	up	0	1
ZNF660	Autism-GSE131706_brain	Corley *et al.*	up	1	1
ZNF681	Autism-GSE131706_brain	Corley *et al.*	up	0	1
ZNF784	Autism-GSE131706_brain	Corley *et al.*	up	1	0
BTNL8	Autism_GSE53924_FRONTAL_CORTEX	Nardone *et al.*	down	0	1
SFMBT1	Autism_GSE53924_FRONTAL_CORTEX	Nardone *et al.*	down	1	0
VTRNA2-1	Autism_GSE53924_FRONTAL_CORTEX	Nardone *et al.*	down	1	1
AKAP12	Autism_GSE53924_FRONTAL_CORTEX	Nardone *et al.*	up	0	1
DAW1	Autism_GSE53924_FRONTAL_CORTEX	Nardone *et al.*	up	0	1
GSTT1	Autism_GSE53924_FRONTAL_CORTEX	Nardone *et al.*	up	0	1
HLA-A	Autism_GSE53924_FRONTAL_CORTEX	Nardone *et al.*	up	1	0
HLA-DQB1	Autism_GSE53924_FRONTAL_CORTEX	Nardone *et al.*	up	0	1
ISOC2	Autism_GSE53924_FRONTAL_CORTEX	Nardone *et al.*	up	1	0
LINC00330	Autism_GSE53924_FRONTAL_CORTEX	Nardone *et al.*	up	0	1
LOC151174	Autism_GSE53924_FRONTAL_CORTEX	Nardone *et al.*	up	0	1
LOC391322	Autism_GSE53924_FRONTAL_CORTEX	Nardone *et al.*	up	0	1
LOC652276	Autism_GSE53924_FRONTAL_CORTEX	Nardone *et al.*	up	0	1
LOC652276	Autism_GSE53924_FRONTAL_CORTEX	Nardone *et al.*	up	0	1
MCCC1	Autism_GSE53924_FRONTAL_CORTEX	Nardone *et al.*	up	0	1
NDUFB9	Autism_GSE53924_FRONTAL_CORTEX	Nardone *et al.*	up	1	0
NKX2-6	Autism_GSE53924_FRONTAL_CORTEX	Nardone *et al.*	up	1	0
NKX6-2	Autism_GSE53924_FRONTAL_CORTEX	Nardone *et al.*	up	1	0
NKX6-2	Autism_GSE53924_FRONTAL_CORTEX	Nardone *et al.*	up	1	0
NR2F2	Autism_GSE53924_FRONTAL_CORTEX	Nardone *et al.*	up	0	1
PRR15	Autism_GSE53924_FRONTAL_CORTEX	Nardone *et al.*	up	0	1
RABL2A	Autism_GSE53924_FRONTAL_CORTEX	Nardone *et al.*	up	1	0
RPL23AP82	Autism_GSE53924_FRONTAL_CORTEX	Nardone *et al.*	up	1	0
SPAG1	Autism_GSE53924_FRONTAL_CORTEX	Nardone *et al.*	up	0	1
TATDN1	Autism_GSE53924_FRONTAL_CORTEX	Nardone *et al.*	up	1	0
TPTEP1	Autism_GSE53924_FRONTAL_CORTEX	Nardone *et al.*	up	0	1
TRIM4	Autism_GSE53924_FRONTAL_CORTEX	Nardone *et al.*	up	0	1
TRMT12	Autism_GSE53924_FRONTAL_CORTEX	Nardone *et al.*	up	0	1
VMO1	Autism_GSE53924_FRONTAL_CORTEX	Nardone *et al.*	up	0	1
ZNF562	Autism_GSE53924_FRONTAL_CORTEX	Nardone *et al.*	up	0	1
ZNF681	Autism_GSE53924_FRONTAL_CORTEX	Nardone *et al.*	up	0	1

Gene Symbol	Dataset	Author	Up/Down Methylated	#Cases	# Controls
WDR88	Autism_GSE53162_Brain	Ladd-Acosta *et al.*	down	0	1
ACTR3C	Autism_GSE53162_Brain	Ladd-Acosta *et al.*	up	0	1
CALCA	Autism-GSE100905_Blood	Kimura *et al.*	up	0	1
EIPR1	Autism-GSE100905_Blood	Kimura *et al.*	up	0	1
FAM193B	Autism-GSE100905_Blood	Kimura *et al.*	up	0	1
BOLL	Autism-GSE113967_blood	Siu *et al.*	down	0	1
KCNAB3	Autism-GSE113967_blood	Siu *et al.*	down	0	1
KCNAB3	Autism-GSE113967_blood	Siu *et al.*	down	0	1
LINC02470	Autism-GSE113967_blood	Siu *et al.*	down	0	1
LOC100507547	Autism-GSE113967_blood	Siu *et al.*	down	0	1
CHMP4C	Autism-GSE113967_blood	Siu *et al.*	up	0	1
PXMP4	Autism-GSE113967_blood	Siu *et al.*	up	0	1
RASSF6	Autism-GSE113967_blood	Siu *et al.*	up	0	1
RNF5	Autism-GSE113967_blood	Siu *et al.*	up	0	1
PRDM13	Autism-GSE131706_brain	Corley *et al.*	up	0	1
BTNL8	Autism_GSE53924_FRONTAL_CORTEX	Nardone *et al.*	down	0	1
TMEM163	Autism-GSE113967_blood	Siu *et al.*	down	0	1
TMEM163	Autism-GSE113967_blood	Siu *et al.*	down	0	1
NKX1-1	Autism-GSE113967_blood	Siu *et al.*	up	0	1
NKX1-1	Autism-GSE113967_blood	Siu *et al.*	up	0	1
NKX1-1	Autism-GSE113967_blood	Siu *et al.*	up	0	1
HLA-DPB2	Autism_GSE53162_Brain	Ladd-Acosta *et al.*	down	1	0
ELOA2	Autism-GSE83424_Blood	Homs *et al.*	down	1	0
TCF19	Autism-GSE83424_Blood	Homs *et al.*	up	1	0
TRIM4	Autism-GSE83424_Blood	Homs *et al.*	up	1	0
FBXL16	Autism-GSE100905_Blood	Kimura *et al.*	up	1	0
NPY	Autism-GSE100905_Blood	Kimura *et al.*	up	1	0
ZNF300P1	Autism-GSE100905_Blood	Kimura *et al.*	up	1	0
EXOC2	Autism-GSE113967_blood	Siu *et al.*	down	1	0
HUS1B	Autism-GSE113967_blood	Siu *et al.*	down	1	0
KATNAL2	Autism-GSE113967_blood	Siu *et al.*	down	1	0
LDHC	Autism-GSE113967_blood	Siu *et al.*	down	1	0
MBP	Autism-GSE113967_blood	Siu *et al.*	down	1	0
AIF1	Autism-GSE113967_blood	Siu *et al.*	up	1	0
CALCB	Autism-GSE113967_blood	Siu *et al.*	up	1	0
CCK	Autism-GSE113967_blood	Siu *et al.*	up	1	0
FAR2	Autism-GSE113967_blood	Siu *et al.*	up	1	0
KATNB1	Autism-GSE113967_blood	Siu *et al.*	up	1	0
LCLAT1	Autism-GSE113967_blood	Siu *et al.*	up	1	0
LINC01341	Autism-GSE113967_blood	Siu *et al.*	up	1	0
SCART1	Autism-GSE113967_blood	Siu *et al.*	up	1	0
SOGA3	Autism-GSE113967_blood	Siu *et al.*	up	1	0
AGPAT1	Autism-GSE131706_brain	Corley *et al.*	up	1	0
ZNF784	Autism-GSE131706_brain	Corley *et al.*	up	1	0
AKAP12	Autism-GSE83424_Blood	Homs *et al.*	up	1	0
CCDC169	Autism-GSE83424_Blood	Homs *et al.*	up	1	0

Gene Symbol	Dataset	Author	Up/Down Methylated	#Cases	# Controls
ELOA2	Autism-GSE113967_blood	Siu *et al.*	down	1	0
CPEB1	Autism-GSE113967_blood	Siu *et al.*	up	1	0
LOC285804	Autism-GSE113967_blood	Siu *et al.*	up	1	0
MCCC1	Autism-GSE113967_blood	Siu *et al.*	up	1	0
NPY	Autism-GSE113967_blood	Siu *et al.*	up	1	0
ZNF300P1	Autism-GSE113967_blood	Siu *et al.*	up	1	0
AKAP12	Autism-GSE83424_Blood	Homs *et al.*	up	1	0
CCDC169	Autism-GSE83424_Blood	Homs *et al.*	up	1	0
ELOA2	Autism-GSE113967_blood	Siu *et al.*	down	1	0
CPEB1	Autism-GSE113967_blood	Siu *et al.*	up	1	0
LOC285804	Autism-GSE113967_blood	Siu *et al.*	up	1	0
MCCC1	Autism-GSE113967_blood	Siu *et al.*	up	1	0
NPY	Autism-GSE113967_blood	Siu *et al.*	up	1	0
ZNF300P1	Autism-GSE113967_blood	Siu *et al.*	up	1	0
BTNL3	Autism_GSE53162_Brain	Ladd-Acosta *et al.*	down	1	1
ERGIC1	Autism_GSE53162_Brain	Ladd-Acosta *et al.*	up	1	1
MARCO	Autism_GSE53162_Brain	Ladd-Acosta *et al.*	up	1	0
KMT2C	Autism_GSE53162_Brain	Ladd-Acosta *et al.*	up	0	1
GSTTP2	Autism_GSE53162_Brain	Ladd-Acosta *et al.*	up	0	1
FAM231D	Autism_GSE53162_Brain	Ladd-Acosta *et al.*	up	0	1
CFAP46	Autism_GSE53162_Brain	Ladd-Acosta *et al.*	up	1	0
GOLGA8B	Autism_GSE53162_Brain	Ladd-Acosta *et al.*	up	1	0
SNORA70J	Autism_GSE53162_Brain	Ladd-Acosta *et al.*	up	1	1
ZNF141	Autism_GSE53162_Brain	Ladd-Acosta *et al.*	up	0	1
LOC154449	Autism-GSE83424_Blood	Homs *et al.*	down	0	1
PEG3	Autism-GSE83424_Blood	Homs *et al.*	down	1	0
ZIM2	Autism-GSE83424_Blood	Homs *et al.*	down	1	0
HLA-F	Autism-GSE83424_Blood	Homs *et al.*	down	1	0
CALHM1	Autism-GSE83424_Blood	Homs *et al.*	up	0	1
CCDC169-SOHLH2	Autism-GSE83424_Blood	Homs *et al.*	up	1	0
HOXA9	Autism-GSE83424_Blood	Homs *et al.*	up	1	0
B4GALNT4	Autism-GSE83424_Blood	Homs *et al.*	up	1	0
LMCD1-AS1	Autism-GSE83424_Blood	Homs *et al.*	up	1	0
MIR1275	Autism-GSE83424_Blood	Homs *et al.*	up	1	0
SP2-AS1	Autism-GSE83424_Blood	Homs *et al.*	up	0	1
SNRNP25	Autism-GSE83424_Blood	Homs *et al.*	up	1	0
RNU5E-1	Autism-GSE83424_Blood	Homs *et al.*	up	1	0
S100A5	Autism-GSE83424_Blood	Homs *et al.*	up	0	1
TRIM40	Autism-GSE83424_Blood	Homs *et al.*	up	0	1
HLA-E	Autism-GSE83424_Blood	Homs *et al.*	up	1	1
USF1	Autism-GSE83424_Blood	Homs *et al.*	up	1	0
APOBEC1	Autism-GSE100905_Blood	Kimura *et al.*	down	0	1
ALLC	Autism-GSE100905_Blood	Kimura *et al.*	down	1	1
HAGH	Autism-GSE100905_Blood	Kimura *et al.*	down	1	1
GLI2	Autism-GSE100905_Blood	Kimura *et al.*	down	1	0

Gene Symbol	Dataset	Author	Up/Down Methylated	#Cases	# Controls
CCDC169-SOHLH2	Autism-GSE100905_Blood	Kimura *et al.*	up	1	1
EML1	Autism-GSE100905_Blood	Kimura *et al.*	up	1	0
RRAGC	Autism-GSE100905_Blood	Kimura *et al.*	up	1	0
FAM53A	Autism-GSE100905_Blood	Kimura *et al.*	up	1	1
LOC644145	Autism-GSE100905_Blood	Kimura *et al.*	up	1	0
PLEKHG4B	Autism-GSE100905_Blood	Kimura *et al.*	up	1	0
OSBP	Autism-GSE100905_Blood	Kimura *et al.*	up	1	0
KEAP1	Autism-GSE100905_Blood	Kimura *et al.*	up	1	1
FH	Autism-GSE100905_Blood	Kimura *et al.*	up	1	0
S100A5	Autism-GSE100905_Blood	Kimura *et al.*	up	1	0
HLA-E	Autism-GSE100905_Blood	Kimura *et al.*	up	1	0
TRIM6-TRIM34	Autism-GSE100905_Blood	Kimura *et al.*	up	0	1
NDUFS2	Autism-GSE113967_blood	Siu *et al.*	down	1	0
FOXCUT	Autism-GSE113967_blood	Siu *et al.*	down	1	0
HAGH	Autism-GSE113967_blood	Siu *et al.*	down	0	1
TRAPPC1	Autism-GSE113967_blood	Siu *et al.*	down	0	1
LINC01114	Autism-GSE113967_blood	Siu *et al.*	down	0	1
LOC154449	Autism-GSE113967_blood	Siu *et al.*	down	1	1
LOC401286	Autism-GSE113967_blood	Siu *et al.*	down	1	0
ZNF696	Autism-GSE113967_blood	Siu *et al.*	down	0	1
MIR219B	Autism-GSE113967_blood	Siu *et al.*	down	1	0
RPUSD1	Autism-GSE113967_blood	Siu *et al.*	down	1	0
LINC01276	Autism-GSE113967_blood	Siu *et al.*	down	0	1
SAMD11	Autism-GSE113967_blood	Siu *et al.*	down	0	1
TEX14	Autism-GSE113967_blood	Siu *et al.*	down	1	0
TRIM40	Autism-GSE113967_blood	Siu *et al.*	down	0	1
MAF1	Autism-GSE113967_blood	Siu *et al.*	down	0	1
NR1H3	Autism-GSE113967_blood	Siu *et al.*	up	1	0
RNF5	Autism-GSE113967_blood	Siu *et al.*	up	1	1
CDKN3	Autism-GSE113967_blood	Siu *et al.*	up	1	0
GOLGA2P11	Autism-GSE113967_blood	Siu *et al.*	up	0	1
CALHM1	Autism-GSE113967_blood	Siu *et al.*	up	1	1
CCDC169-SOHLH2	Autism-GSE113967_blood	Siu *et al.*	up	1	1
ST6GAL2	Autism-GSE113967_blood	Siu *et al.*	up	1	1
CKLF-CMTM1	Autism-GSE113967_blood	Siu *et al.*	up	1	1
TYW3	Autism-GSE113967_blood	Siu *et al.*	up	1	1
SLC45A4	Autism-GSE113967_blood	Siu *et al.*	up	1	1
NSUN3	Autism-GSE113967_blood	Siu *et al.*	up	0	1
LINC01230	Autism-GSE113967_blood	Siu *et al.*	up	1	0
DUOXA2	Autism-GSE113967_blood	Siu *et al.*	up	1	0
NDUFAF2	Autism-GSE113967_blood	Siu *et al.*	up	1	0
CLDND2	Autism-GSE113967_blood	Siu *et al.*	up	1	0
CADPS2	Autism-GSE113967_blood	Siu *et al.*	up	0	1
LOC101927248	Autism-GSE113967_blood	Siu *et al.*	up	1	1
GTF2I	Autism-GSE113967_blood	Siu *et al.*	up	1	0

Gene Symbol	Dataset	Author	Up/Down Methylated	#Cases	# Controls
OR2B2	Autism-GSE113967_blood	Siu *et al.*	up	1	1
PUS7L	Autism-GSE113967_blood	Siu *et al.*	up	0	1
HCG15	Autism-GSE113967_blood	Siu *et al.*	up	1	0
RPN1	Autism-GSE113967_blood	Siu *et al.*	up	0	1
LOC101927560	Autism-GSE113967_blood	Siu *et al.*	up	1	1
EVX2	Autism-GSE113967_blood	Siu *et al.*	up	1	1
SAMD11	Autism-GSE113967_blood	Siu *et al.*	up	1	0
TRAF3IP3	Autism-GSE113967_blood	Siu *et al.*	up	0	1
OR6S1	Autism-GSE113967_blood	Siu *et al.*	up	1	0
PDPK1	Autism-GSE113967_blood	Siu *et al.*	up	1	1
RAD17	Autism-GSE113967_blood	Siu *et al.*	up	1	0
ADIPOR2	Autism-GSE113967_blood	Siu *et al.*	up	0	1
DHX37	Autism-GSE113967_blood	Siu *et al.*	up	1	0
FAM53A	Autism-GSE113967_blood	Siu *et al.*	up	0	1
MRPS34	Autism-GSE113967_blood	Siu *et al.*	up	1	0
OSBP	Autism-GSE113967_blood	Siu *et al.*	up	1	0
KEAP1	Autism-GSE113967_blood	Siu *et al.*	up	1	0
KLHL17	Autism-GSE113967_blood	Siu *et al.*	up	1	0
SP2-AS1	Autism-GSE113967_blood	Siu *et al.*	up	1	1
PRDM15	Autism-GSE113967_blood	Siu *et al.*	up	0	1
RNF39	Autism-GSE113967_blood	Siu *et al.*	up	1	1
CYP2E1	Autism-GSE113967_blood	Siu *et al.*	up	1	0
FLJ32255	Autism-GSE113967_blood	Siu *et al.*	up	1	0
HINT2	Autism-GSE113967_blood	Siu *et al.*	up	0	1
TMEM106B	Autism-GSE113967_blood	Siu *et al.*	up	1	0
PARK7	Autism-GSE113967_blood	Siu *et al.*	up	0	1
SH3BP2	Autism-GSE113967_blood	Siu *et al.*	up	1	0
HLA-E	Autism-GSE113967_blood	Siu *et al.*	up	1	0
TRIM6-TRIM34	Autism-GSE113967_blood	Siu *et al.*	up	1	1
HARS2	Autism-GSE113967_blood	Siu *et al.*	up	0	1
ZNF559-ZNF177	Autism-GSE113967_blood	Siu *et al.*	up	1	0
LINC01012	Autism-GSE113967_blood	Siu *et al.*	up	1	0
ZNF561-AS1	Autism-GSE113967_blood	Siu *et al.*	up	1	1
VTRNA2-1	Autism-GSE131706_brain	Corley *et al.*	down	1	0
RNF5	Autism-GSE131706_brain	Corley *et al.*	up	1	0
CCDC169-SOHLH2	Autism-GSE131706_brain	Corley *et al.*	up	1	0
FRG2	Autism-GSE131706_brain	Corley *et al.*	up	0	1
ABHD16A	Autism-GSE131706_brain	Corley *et al.*	up	1	1
LOC101928306	Autism-GSE131706_brain	Corley *et al.*	up	1	1
RNF39	Autism-GSE131706_brain	Corley *et al.*	up	1	1
MYMX	Autism-GSE131706_brain	Corley *et al.*	up	0	1
TNP2	Autism-GSE131706_brain	Corley *et al.*	up	1	1
CCNA1	Autism-GSE131706_brain	Corley *et al.*	up	0	1
TRIM6-TRIM34	Autism-GSE131706_brain	Corley *et al.*	up	1	0
HDDC3	Autism-GSE131706_brain	Corley *et al.*	up	1	0

Gene Symbol	Dataset	Author	Up/Down Methylated	#Cases	# Controls
ZNF660-ZNF197	Autism-GSE131706_brain	Corley *et al.*	up	1	1
BTNL3	Autism_GSE53924_FRONTAL_CORTEX	Nardone *et al.*	down	0	1
NUFIP1	Autism_GSE53924_FRONTAL_CORTEX	Nardone *et al.*	up	0	1
PDPK1	Autism_GSE53924_FRONTAL_CORTEX	Nardone *et al.*	up	0	1
CFAP46	Autism_GSE53924_FRONTAL_CORTEX	Nardone *et al.*	up	1	0
SPATA8-AS1	Autism_GSE53924_FRONTAL_CORTEX	Nardone *et al.*	up	0	1
TRAPPC1	Autism-GSE113967_blood	Siu *et al.*	down	0	1
BTNL3	Autism_GSE53924_FRONTAL_CORTEX	Nardone *et al.*	down	0	1
FAM53A	Autism-GSE113967_blood	Siu *et al.*	up	0	1
FAM53A	Autism-GSE113967_blood	Siu *et al.*	up	0	1
FAM53A	Autism-GSE113967_blood	Siu *et al.*	up	0	1
CYP2E1	Autism-GSE113967_blood	Siu *et al.*	up	1	0
CCDC169-SOHLH2	Autism-GSE83424_Blood	Homs *et al.*	up	1	0
CCDC169-SOHLH2	Autism-GSE83424_Blood	Homs *et al.*	up	1	0
ZIM2	Autism-GSE83424_Blood	Homs *et al.*	down	1	0
RNF5P1	Autism-GSE113967_blood	Siu *et al.*	up	1	1
CMTM1	Autism-GSE113967_blood	Siu *et al.*	up	1	1
RNF5P1	Autism-GSE131706_brain	Corley *et al.*	up	1	0

Supplementary Table 7. Characteristics of genes affected by rare epivariations.

Gene	Function	Associated with ASD?	Expressed in the Central Nervous System?	Notes	Epivariation Status
Neuropeptide Y (NPY)	Cortical excitability; stress response, food intake; circadian rhythms; pain perception; cardiovascular function; antimicrobial activity	No	Yes	Altered plasma NPY level in ASD (1)	Hypermethylated
NK6 Homeobox 2 (NKX6-2)	Cell differentiation; regulation of myelination; neurogenesis; neuromuscular process controlling balance.	No	Yes	Hypermethylated after lead exposure in mother's blood (2)	Hypermethylated
1-Acylglycerol-3-Phosphate O-Acyltransferase 1 (AGPAT1)	Signal transduction; lipid biosynthes is in cells	No	Yes	Implicated in neurodegenerative diseases (3)	Hypermethylated
Katanin Regulatory Subunit B1 (KATNB1)	Regulation of neuronal dendritic arborization	No	Yes	Associated with lissencephaly and microcephaly (4, 5)	Hypermethylated
A-Kinase Anchoring Protein 12 (AKAP12)	Blood-retinal and blood-brain barriers formation	No	Yes (overexpressed)	AKAP8 is associated with ASD (6)	Hypermethylated
Fatty Acyl-CoA Reductase 2 (FAR2)	Wax and plasmalogen biosynthesis	No	Yes	FAR1 mutations are connected to a syndrome with intellectual disability and epilepsy (7, 8)	Hypermethylated
Leucine Rich Repeat And Ig Domain Containing 3 (LINGO3)	Component of cell membrane, active in extracellular matrix	No	Yes (overexpressed)	LINGO2 loss CNV has been associated with ASD (9); LINGO3 stress-induced epigenetic mutation are associated with depression (10)	Hypermethylated
Tripartite Motif Containing 4 (TRIM4)	Not identified yet	No	Yes	TRIM4 is hypomethylated in subjects with neural tube defects (11)	Hypermethylated
Cytoplasmic Polyadenylation Element Binding Protein 1 (CPEB1)	Regulation of mRNA translation; processing of the 3' untranslated region; cell proliferation; tumorigenesis	Yes (in Fragile X syndrome) (12)	Yes (overexpressed)	CPEB4 has been associated with ASD (13)	Hypermethylated
Methylcrotonyl-CoA Carboxylase Subunit 1 (MCCC1)	Mitochondrial enzyme; leucine catabolism	No	Yes	MCCC1 variants are associated with Parkinson's Disease (14)	Hypermethylated
Elongin A2 (ELOA2)	RNA chain elongation	No	Ubiquitously expressed	Imprinted gene (15)	Hypomethylated
Asteroid Homolog 1 (ASTE1)	Enable nuclease activity	No	Yes	-	Hypermethylated
Testis Expressed 14, Intercellular Bridge Forming Factor (TEX14)	Spermatogenesis	No	Yes (overexpressed in fetal brain)	-	Hypomethylated
EF-Hand Calcium Binding Domain 10 (EFCAB10)	Enable calcium ion binding activity	No	Yes	-	Hypermethylated
Lactate Dehydrogenase C (LDHC)	Anaerobic glycolysis	No	Yes	-	Hypomethylated
TRIM39-RPP21 Readthrough (TRIM39-RPP21)	Mediation of interferon response	No	Yes	-	Hypermethylated
Olfactory Receptor Family 4 Subfamily D Member 9 (OR4D9)	Recognition and G protein-mediated transduction of odorant signals	No	Yes	-	Hypermethylated

Gene	Function	Associated with ASD?	Expressed in the Central Nervous System?	Notes	Epivariation Status
Zinc Finger Protein 300 Pseudogene 1 (ZNF300P1)	Embrionyc development	No	Yes	Pseudogene	Hypermethylated
Oxysterol Binding Protein (OSBP)	Transportation of sterols from lysosomes to the nucleus	No	Yes	OSBP expression decreased during the development of mouse cortex, OSBP overexpression reduces neurite growth (16)	Hypermethylated
Major Histocompatibility Complex, Class I, E (HLA-E)	Involved in immune self-nonself discrimination	No	Yes	Absence of association with ASD was reported in a cohort of only 15 children (17)	Hypermethylated
Scm Like With Four Mbt Domains 1 (SFMBT1)	Involved in antigen recognition, regulation of neurodevelopment and dendritic spine density	No	Yes	SFMBT1 copy number loss is associated with idiopathic normal pressure hydrocephalus (18). SFMBT1 expression is associated with schizophrenia (19)	Hypomethylated
Cilia And Flagella Associated Protein 46 (CFAP46)	Involved in axoneme assembly	No	Yes	Decreased expression of CFAP46 in patients with panic disorder who respond to psychotherapy (20)	Hypermethylated
Glutamate Ionotropic Receptor Kainate Type Subunit 2 (GRIK2)	Glutamate transmission and neuronal growth	Yes (21, 22)	Yes (overexpressed)	Not associated with ASD in the Indian and Chinese populations (23, 24)	Hypermethylated
Spermatogenesis And Oogenesis Specific Basic Helix-Loop-Helix 2 (SOHLH2)	Spermatogenesis, oogenesis and folliculogenesis	No	Yes	-	-
Farnesyl-Diphosphate Farnesyltransferase 1 (FDFT1)	Cholesterol biosynthesis	No	Yes	FDFT1 is overexpressed after prolonged cocaine, cannabis and phencyclidine abuse (25)	Hypermethylated
Small Nucleolar RNA Host Gene 32 (SNHG32 or C6orf48)	Enables double-stranded RNA binding activity	No	Yes	-	Hypermethylated
WNK Lysine Deficient Protein Kinase 4 (WNK4)	Regulation of several types of ion channels, cotransporters, and exchangers involved in electrolyte flux	No	Yes	WNK3 deletion was associated with ASD (26)	Hypermethylated
Phospholipase D Family Member 6 (PLD6)	Involved in mitochondrial fusion	No	Yes	-	Hypermethylated
Coiled-Coil Domain Containing 169 (CCDC169)	Sperm motility	No	Yes	-	Hypermethylated
Cholecystokinin (CCK)	Regulation of gastric acid secretion and food intake; modulation of neuronal activity in the brain	Yes (27)	Yes (overexpressed)	Cholecystokinin levels in mononuclear blood cells are not different between ASD and controls (28)	Hypermethylated
Ribosomal Protein L13 (RPL13)	Encodes a ribosomal protein that is a component of the 60S subunit	No	Yes	RPL13 is associated with Alzheimer's Disease (29)	Hypermethylated
NADH:Ubiquinone Oxidoreductase Subunit B9 (NDUFB9)	Subunit of the mitochondrial oxidative phosphorylation complex I	No	Yes (overexpressed)	NDUFB9 is associated with cocaine dependance (30)	Hypermethylated
Brevican (BCAN)	Specifically expressed in the central nervous system, regulates the formation of the brain extracellular matrix	No	Yes (overexpressed)	BCAN cerebrospinal fluid levels are associated to Alzheimer's disease (31)	Hypermethylated
Endothelin Receptor Type B (EDNRB)	Encodes a G protein-coupled, seven-transmembrane domain	No	Yes	-	Hypermethylated

Gene	Function	Associated with ASD?	Expressed in the Central Nervous System?	Notes	Epivariation Status
	receptor that recognizes a family of vasoactive peptides known as endothelins				
Potassium Voltage-Gated Channel Subfamily A Regulatory Beta Subunit 2 (KCNAB2)	Regulation of neurotransmitter release, heart rate, neuronal excitability, epithelial electrolyte transport, smooth muscle contraction, and cell volume	No	Yes (overexpressed)	KCNAB2 is associated with epilepsy (32)	Hypermethylated
Catechol-O-Methyltransferase (COMT)	Catecholamine degradation pathway	Yes (33)	Yes	Inconsistent findings (34, 35)	Hypermethylated
Kelch Like ECH Associated Protein 1 (KEAP1)	Regulation of translational control and Class I MHC mediated antigen processing and presentation	Yes	Yes	KEAP1 serum levels are higher in ASD (36)	Hypermethylated
Fumarate Hydratase (FH)	Conversion of fumarate in L-malate	Yes (37)	Yes	Only one case report (37)	Hypermethylated
Ring Finger Protein 5 (RNF5)	Regulation of cell motility and cell adhesion	No	Yes (overexpressed)	Differentially methylated in Parkinson's disease and in schizophrenia (3, 38)	Hypermethylated
NADH:Ubiquinone Oxidoreductase Core Subunit S2 (NDUFS2)	Core subunit of the mitochondrial membrane respiratory chain NADH dehydrogenase (Complex I)	No	Yes	-	Hypomethylated
Succinate Dehydrogenase Complex Flavoprotein Subunit A (SDHA)	Catalytic subunit of succinate-ubiquinone oxidoreductase, complex of the mitochondrial respiratory chain	No	Yes	Altered expression in schizophrenia and major depression (39, 40)	Hypermethylated

REFERENCES

[1] Çelikkol Sadıç Ç, Bilgiç A, Kılınç İ, Oflaz MB, Baysal T. Evaluation of Appetite-Regulating Hormones ın Young Children with Autism Spectrum Disorder. Journal of autism and developmental disorders. 2021; 51(2): 632-43.

[2] Martínez-Pacheco M, Hidalgo-Miranda A, Romero-Córdoba S, Valverde M, Rojas E. MRNA and miRNA expression patterns associated to pathways linked to metal mixture health effects. Gene. 2014; 533(2): 508-14.

[3] Henderson AR, Wang Q, Meechoovet B, Siniard AL, Naymik M, De Both M, *et al.* DNA Methylation and Expression Profiles of Whole Blood in Parkinson's Disease. Front Genet. 2021; 12: 640266.

[4] Mishra-Gorur K, Caglayan AO, Schaffer AE, Chabu C, Henegariu O, Vonhoff F, *et al.* Mutations in KATNB1 Cause Complex Cerebral Malformations by Disrupting Asymmetrically Dividing Neural Progenitors. Neuron. 2015; 85(1): 228.

[5] Yigit G, Wieczorek D, Bogershausen N, Beleggia F, Moller-Hartmann C, Altmuller J, *et al.* A syndrome of microcephaly, short stature, polysyndactyly, and dental anomalies caused by a homozygous KATNB1 mutation. Am J Med Genet A. 2016; 170(3): 728-33.

[6] Poelmans G, Franke B, Pauls DL, Glennon JC, Buitelaar JK. AKAPs integrate genetic findings for autism spectrum disorders. Transl Psychiatry. 2013; 3: e270.

[7] Chen J, Wu W, Fu Y, Yu S, Cui D, Zhao M, *et al.* Increased expression of fatty acid synthase and acetyl-CoA carboxylase in the prefrontal cortex and cerebellum in the valproic acid model of autism. Exp Ther Med. 2016; 12(3): 1293-8.

[8] Alshenaifi J, Ewida N, Anazi S, Shamseldin HE, Patel N, Maddirevula S, *et al.* The many faces of peroxisomal disorders: Lessons from a large Arab cohort. Clin Genet. 2019; 95(2): 310-9.

[9] Matsunami N, Hensel CH, Baird L, Stevens J, Otterud B, Leppert T, *et al.* Identification of rare DNA sequence variants in high-risk autism families and their prevalence in a large case/control population. Mol Autism. 2014; 5(1): 5.

[10] Park C, Rosenblat JD, Brietzke E, Pan Z, Lee Y, Cao B, *et al.* Stress, epigenetics and depression: A systematic review. Neuroscience and biobehavioral reviews. 2019; 102: 139-52.

[11] Zhang H, Guo Y, Gu H, Wei X, Ma W, Liu D, *et al.* TRIM4 is associated with neural tube defects based on genome-wide DNA methylation analysis. Clin Epigenetics. 2019; 11(1): 17.

[12] Udagawa T, Farny NG, Jakovcevski M, Kaphzan H, Alarcon JM, Anilkumar S, *et al.* Genetic and acute CPEB1 depletion ameliorate fragile X pathophysiology. Nat Med. 2013; 19(11): 1473-7.

[13] Parras A, Anta H, Santos-Galindo M, Swarup V, Elorza A, Nieto-Gonzalez JL, *et al.* Autism-like phenotype and risk gene mRNA deadenylation by CPEB4 mis-splicing. Nature. 2018; 560(7719): 441-6.

[14] Foo JN, Tan LC, Irwan ID, Au WL, Low HQ, Prakash KM, *et al.* Genome-wide association study of Parkinson's disease in East Asians. Hum Mol Genet. 2017; 26(1): 226-32.

[15] Strichman-Almashanu LZ, Lee RS, Onyango PO, Perlman E, Flam F, Frieman MB, *et al.* A genome-wide screen for normally methylated human CpG islands that can identify novel imprinted genes. Genome Res. 2002; 12(4): 543-54.

[16] Gu X, Li A, Liu S, Lin L, Xu S, Zhang P, *et al.* MicroRNA124 Regulated Neurite Elongation by Targeting OSBP. Mol Neurobiol. 2016; 53(9): 6388-96.

[17] Kharrat N, Abdelhedi R, Gtif I, Ayadi I, Rizzo R, Bortolotti D, *et al.* Non-classical human leukocyte antigen class I in Tunisian children with autism. Cent Eur J Immunol. 2020; 45(2): 176-83.
[18] Korhonen VE, Helisalmi S, Jokinen A, Jokinen I, Lehtola JM, Oinas M, *et al.* Copy number loss in SFMBT1 is common among Finnish and Norwegian patients with iNPH. Neurology Genetics. 2018; 4(6): e291.
[19] Li Y, Ma C, Li S, Wang J, Li W, Yang Y, *et al.* Regulatory Variant rs2535629 in ITIH3 Intron Confers Schizophrenia Risk By Regulating CTCF Binding and SFMBT1 Expression. Adv Sci (Weinh). 2022; 9(6): e2104786.
[20] Ziegler C, Grundner-Culemann F, Schiele MA, Schlosser P, Kollert L, Mahr M, *et al.* The DNA methylome in panic disorder: a case-control and longitudinal psychotherapy-epigenetic study. Transl Psychiatry. 2019; 9(1): 314.
[21] Kim SA, Kim JH, Park M, Cho IH, Yoo HJ. Family-based association study between GRIK2 polymorphisms and autism spectrum disorders in the Korean trios. Neurosci Res. 2007; 58(3): 332-5.
[22] Leblond CS, Cliquet F, Carton C, Huguet G, Mathieu A, Kergrohen T, *et al.* Both rare and common genetic variants contribute to autism in the Faroe Islands. NPJ Genom Med. 2019; 4: 1.
[23] Xie X, Hou F, Li L, Chen Y, Liu L, Luo X, *et al.* Polymorphisms of Ionotropic Glutamate Receptor-Related Genes and the Risk of Autism Spectrum Disorder in a Chinese Population. Psychiatry Investig. 2019; 16(5): 379-85.
[24] Dutta S, Das S, Guhathakurta S, Sen B, Sinha S, Chatterjee A, *et al.* Glutamate receptor 6 gene (GluR6 or GRIK2) polymorphisms in the Indian population: a genetic association study on autism spectrum disorder. Cell Mol Neurobiol. 2007; 27(8): 1035-47.
[25] Lehrmann E, Colantuoni C, Deep-Soboslay A, Becker KG, Lowe R, Huestis MA, *et al.* Transcriptional changes common to human cocaine, cannabis and phencyclidine abuse. PLoS One. 2006; 1: e114.
[26] Qiao Y, Liu X, Harvard C, Hildebrand MJ, Rajcan-Separovic E, Holden JJ, *et al.* Autism-associated familial microdeletion of Xp11.22. Clin Genet. 2008; 74(2): 134-44.
[27] Iourov IY, Vorsanova SG, Voinova VY, Yurov YB. 3p22.1p21.31 microdeletion identifies CCK as Asperger syndrome candidate gene and shows the way for therapeutic strategies in chromosome imbalances. Mol Cytogenet. 2015; 8: 82.
[28] Brambilla F, Guareschi-Cazzullo A, Tacchini C, Musetti C, Panerai AE, Sacerdote P. Beta-endorphin and cholecystokinin 8 concentrations in peripheral blood mononuclear cells of autistic children. Neuropsychobiology. 1997; 35(1): 1-4.
[29] De Jager PL, Srivastava G, Lunnon K, Burgess J, Schalkwyk LC, Yu L, *et al.* Alzheimer's disease: early alterations in brain DNA methylation at ANK1, BIN1, RHBDF2 and other loci. Nat Neurosci. 2014; 17(9): 1156-63.
[30] Huggett SB, Stallings MC. Genetic Architecture and Molecular Neuropathology of Human Cocaine Addiction. J Neurosci. 2020; 40(27): 5300-13.
[31] Minta K, Brinkmalm G, Portelius E, Johansson P, Svensson J, Kettunen P, *et al.* Brevican and Neurocan Peptides as Potential Cerebrospinal Fluid Biomarkers for Differentiation Between Vascular Dementia and Alzheimer's Disease. J Alzheimers Dis. 2021; 79(2): 729-41.
[32] Heilstedt HA, Burgess DL, Anderson AE, Chedrawi A, Tharp B, Lee O, *et al.* Loss of the potassium channel beta-subunit gene, KCNAB2, is associated with epilepsy in patients with 1p36 deletion syndrome. Epilepsia. 2001; 42(9): 1103-11.
[33] Esmaiel NN, Ashaat EA, Mosaad R, Fayez A, Ibrahim M, Abdallah ZY, *et al.* The potential impact of COMT gene variants on dopamine regulation and phenotypic traits of ASD patients. Behav Brain Res. 2020; 378: 112272.
[34] Limprasert P, Maisrikhaw W, Sripo T, Wirojanan J, Hansakunachai T, Roongpraiwan R, *et al.* No association of Val158Met variant in the COMT gene with autism spectrum disorder in Thai children. Psychiatr Genet. 2014; 24(5): 230-1.
[35] Yang PY, Menga YJ, Li T, Huang Y. Associations of endocrine stress-related gene polymorphisms with risk of autism spectrum disorders: Evidence from an integrated meta-analysis. Autism Res. 2017; 10(11): 1722-36.
[36] Ayaydin H, Akaltun I, Koyuncu I, CelIk H, A KI, Takatak H. High KEAP1, NRF2 and Low HO-1 Serum Levels in Children with Autism. Noro Psikiyatr Ars. 2020; 57(4): 274-9.
[37] Duong BT, Savarirayan R, Winship I. Incidental diagnosis of HLRCC following investigation for Asperger Syndrome: actionable and actioned. Fam Cancer. 2016; 15(1): 25-9.
[38] de Jong S, van Eijk KR, Zeegers DW, Strengman E, Janson E, Veldink JH, *et al.* Expression QTL analysis of top loci from GWAS meta-analysis highlights additional schizophrenia candidate genes. Eur J Hum Genet. 2012; 20(9): 1004-8.
[39] Weickert CS, Sheedy D, Rothmond DA, Dedova I, Fung S, Garrick T, *et al.* Selection of reference gene expression in a schizophrenia brain cohort. Aust N Z J Psychiatry. 2010; 44(1): 59-70.
[40] Qi YJ, Lu YR, Shi LG, Demmers JAA, Bezstarosti K, Rijkers E, *et al.* Distinct proteomic profiles in prefrontal subareas of elderly major depressive disorder and bipolar disorder patients. Transl Psychiatry. 2022; 12(1): 275.
